# Transfer learning on protein language models improves antimicrobial peptide classification

**DOI:** 10.1038/s41598-025-21223-y

**Published:** 2025-10-27

**Authors:** Elias Georgoulis, Michaela Areti Zervou, Yannis Pantazis

**Affiliations:** 1 Institute of Applied and Computational Mathematics, FORTH, Heraklion, 700 13 Greece; 2 Department of Mathematics & Applied Mathematics, University of Crete, Heraklion, 700 13 Greece; 3 Department of Computer Science, University of Crete, Heraklion, 700 13 Greece; 4 Institute of Computer Science, FORTH, Heraklion, 700 13 Greece

**Keywords:** Protein language models, Transfer learning, Low-rank adaptation, Antimicrobial peptide classification, Computational protein engineering, Computational biology and bioinformatics, Mathematics and computing

## Abstract

Antimicrobial peptides (AMPs) are essential components of the innate immune system in humans and other organisms, exhibiting potent activity against a broad spectrum of pathogens. Their potential therapeutic applications, particularly in combating antibiotic resistance, have rendered AMP classification a vital task in computational biology. However, the scarcity of labeled AMP sequences, coupled with the diversity and complexity of AMPs, poses significant challenges for the training of standalone AMP classifiers. Self-supervised learning has emerged as a powerful paradigm in addressing such challenges across various fields, leading to the development of Protein Language Models (PLMs). These models leverage vast amounts of unlabeled protein sequences to learn biologically relevant features, providing transferable protein sequence representations (embeddings), that can be fine-tuned for downstream tasks even with limited labeled data. This study evaluates the performance of several publicly-available PLMs in AMP classification utilizing transfer learning techniques and benchmarking them against state-of-the-art neural-based classifiers. Our key findings include: (a) Model scale is crucial, with classification performance consistently improving with increasing model size; (b) State-of-the-art results are achieved with minimal effort utilizing PLM embedding representations alongside shallow classifiers; and (c) Classification performance is further enhanced through efficient fine-tuning of PLMs’ parameters. Code showcasing our pipelines is available at https://github.com/EliasGeorg/PLM_AMP_Classification.

## Introduction

Antimicrobial peptides (AMPs) are naturally occurring peptides found in a wide range of living organisms. They serve as a critical line of defense in the innate immune system against pathogens^1,2^. With their broad-spectrum antimicrobial activity, AMPs have garnered attention as promising candidates for therapeutic applications, particularly in addressing the global challenge of antibiotic resistance^3^. In addition to their antimicrobial role, many AMPs have also demonstrated selective activity against cancer cells^1,4^, further expanding their potential for therapeutic development. However, AMP discovery and characterization remain difficult due to their sequence diversity, limited availability in public databases (only a few thousand are known), and intricate structure-function relationships^1^. AMP classification has predominantly relied on supervised learning, including approaches that either leverage manually curated features^5–13^ or sequence-based neural networks^14–19^. While these methods have achieved reasonable success, they are often hindered by the *scarcity of labeled AMP data*, leading to issues such as overfitting and suboptimal generalization^20,21^. Additionally, these methods typically rely on complex architectures and training procedures that are challenging to implement and require advanced expertise.

Another challenge, distinct from the limitations of supervised approaches, is how to effectively leverage the vast amounts of publicly-available protein sequences while capturing their inherent complexity –a problem that has been recently addressed through self-supervised learning. Self-supervised learning represents a significant paradigm shift in deep learning, enabling researchers to extract valuable insights from large, unlabeled datasets. Largely inspired by the success of Large Language Models (LLMs) in natural language processing^22–28^, Protein Language Models (PLMs)^29–34^ have been established as powerful tools in AI-driven protein research. These models are trained on large-scale protein datasets using substantial computational resources, and self-supervised objectives in which PLMs learn to predict randomly masked amino acids within a sequence based on their surrounding context. This training process allows PLMs to effectively model the protein landscape by learning the implicit evolutionary rules that govern protein sequences, which inherently encode key structural, biochemical, and functional information, thereby producing meaningful protein representations. Like LLMs, which have demonstrated proficiency as few-shot learners through transfer learning techniques^22–26^, PLMs have shown similar few-shot learning capabilities achieving state-of-the-art performance across a variety of tasks, including protein structure prediction^32,33^, function annotation^29,35,36^, and de novo protein design^34,37^.

This study conducts an extensive comparative analysis of various publicly-available PLMs^29,30,32,38–41^, focusing on their performance in AMP classification through transfer learning techniques. We implement and evaluate two primary transfer learning approaches: embedding-based transfer learning, which integrates PLM-generated embeddings with shallow classifiers, and parameter fine-tuning, which efficiently adapts the weights of the PLM to the specific task. Utilizing multiple AMP datasets, we thoroughly evaluate a series of pre-trained PLMs aiming to address the following questions:

(i) Which PLMs deliver the most accurate predictions for the AMP classification task?
(ii) Which characteristics of the PLMs highly correlate with performance metrics?
(iii) How does transfer learning on PLMs compares with state-of-the-art standalone neural-based AMP classifiers^8,9,11,12,14^?
(iv) Can efficient fine-tuning of PLMs’ parameters further enhance performance?

Our findings demonstrate that transfer learning on PLMs consistently outperforms current state-of-the-art models for AMP classification, even with minimal fine-tuning and computational resources. This establishes a robust, scalable, and user-friendly framework for AMP classification, reinforcing the use of PLMs as transformative tools for advancing AI-driven protein research.

## Methods

**Fig. 1:**
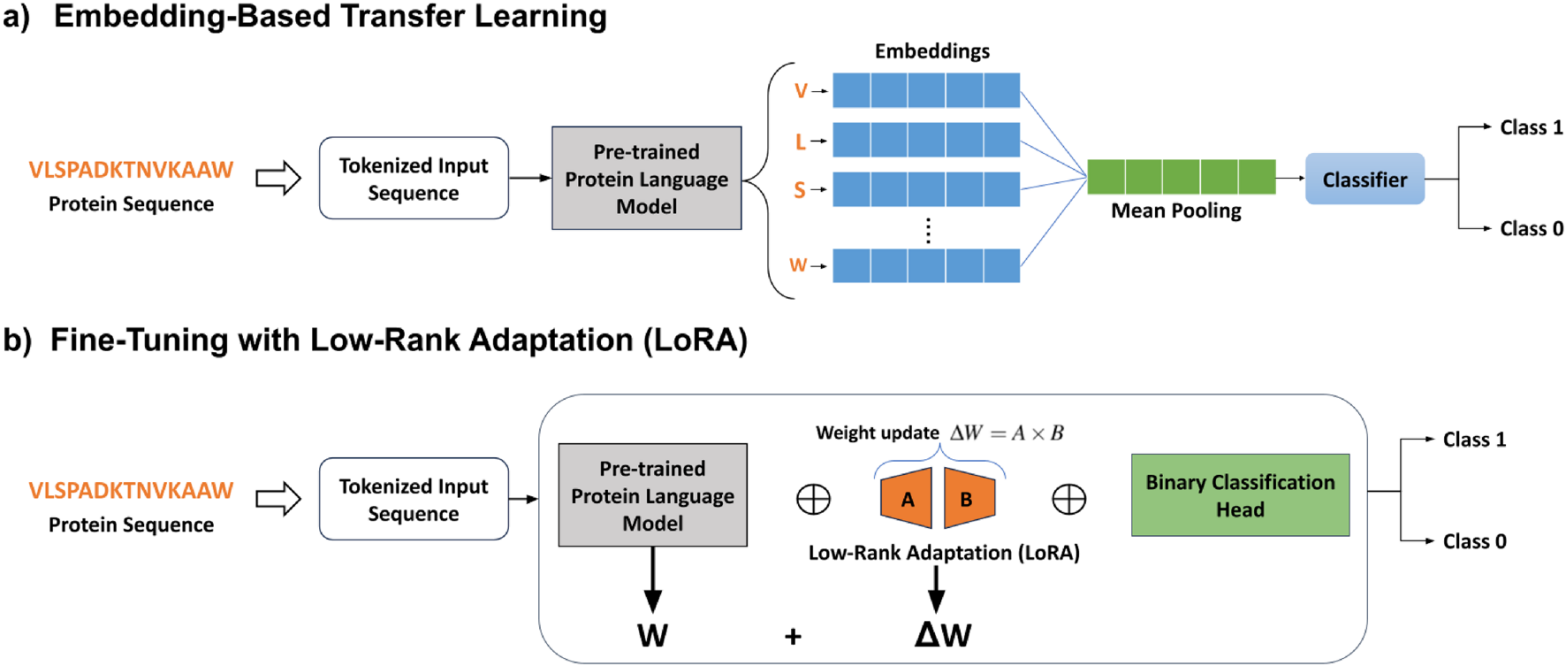
Illustration of the transfer learning pipelines. (**a**) Protein sequences are initially fed into a pre-trained PLM to generate embeddings for each amino acid within the sequence. To obtain fixed-size representations for each sequence, the mean of these embeddings across the sequence length dimension is computed. These embeddings, along with their corresponding protein class labels, are used to train a classifier to perform AMP classification. (**b**) A binary classification head is appended to the final layer of the PLM’s encoder. New trainable parameters ($\Delta W$) are introduced in a parameter-efficient manner called Low-Rank Adaptation (LoRA). These parameters are trained to adapt the PLM specifically for the AMP classification task.

Two distinct transfer learning pipelines were implemented to leverage the capacity of PLMs for the task of AMP classification: an embedding-based approach and a parameter fine-tuning approach. Figure 1 demonstrates the two pipelines. The upper diagram shows the embedding-based approach, where the pre-trained model generates fixed-size vectors for input sequences, which are subsequently used as features for a separate, trainable classifier. The lower diagram illustrates the fine-tuning approach, where the PLM’s weights are fine-tuned by updating them through an efficient adaptation mechanism, allowing the model to learn specific traits related to the AMP classification task.

### Protein language models

PLMs constitute a class of deep neural networks trained on massive amounts of protein sequence data, employing self-supervision at a large scale. This study evaluates the representational capabilities of publicly-available, pre-trained PLMs enlisted in Table 1 with respect to their performance in AMP classification. The selection of PLMs for our comparative analysis was guided by criteria designed to ensure an overarching, fair, and reproducible evaluation. Models were chosen to represent a broad range of parameter sizes (8M to 15B), architectural paradigms (including Transformer-based and recurrent networks), and training datasets (e.g., UniRef, BFD, Pfam). We excluded models requiring multiple sequence alignment (MSA), as they rely on additional evolutionary information not uniformly available across all sequences and introduce a layer of preprocessing that limits scalability and comparability. Only publicly accessible models were considered to support reproducibility. The examined PLMs reflect the prevailing trend in sequence-to-sequence modeling, characterized by the dominance of the Transformer architecture and its associated self-attention mechanism^42^. This mechanism enables the models to process sequences in an adaptive, non-linear and context-sensitive manner, which could be particularly advantageous for deciphering the complex interactions between amino acids. Indeed, the transformer architecture has consistently excelled at identifying long-range dependencies within sequences.

**Table 1:** Overview of the studied PLMs. The table lists key characteristics of the 18 PLMs, including their embedding size, training datasets, architecture details, and the number of parameters.

Model		Embedding size	Training dataset	Number of layers	Model type	Number of parameters
ESM2^32^	t48	5120	UniRef50++	48	Transformer	15B
	t36	2560	UniRef50++	36	Transformer	3B
	t33	1280	UniRef50++	33	Transformer	650M
	t12	480	UniRef50++	12	Transformer	35M
	t6	320	UniRef50++	6	Transformer	8M
ESM	1b^29^	1280	UniRef50	33	Transformer	650M
	1v^38^	1280	UniRef90	33	Transformer	650M
ProtT5^30^	xxl	1024	BFD100/UniRef50	24	Transformer	11B
	xl	1024	BFD100/UniRef50	24	Transformer	3B
	xl-bfd	1024	BFD100	24	Transformer	3B
ProtBert^30^		1024	UniRef100	30	Transformer	420M
ProtBert-bfd^30^		1024	BFD100	30	Transformer	420M
ProtAlbert^30^		4096	UniRef100	12	Transformer	224M
ProtXLNet^30^		1024	UniRef100	30	Transformer	409M
ProteinBert^39^		1562	UniRef90	12	Transformer	16M
TAPE Bert^40^		768	Pfam	12	Transformer	92 M
TAPE Babbler^40^		1900	Pfam	2	mLSTM	18M
SeqVec^41^		1024	UniRef50	2	ELMo	93M

In this work, we explore various models from the Evolutionary Scale Modeling (ESM)^29^ family and its sequel, ESM2^32^. While ESM2 retains core features of ESM, it introduces improvements in both architecture and training process. ESMs are trained on evolutionary-scale data utilizing a variant of the Bidirectional Encoder Representations from Transformers^43^ (BERT) architecture, which is an *encoder-only* architecture with a masked language modeling loss function. In masked language modeling, a certain percentage of tokens from the input sequence are randomly masked, and the model is trained to predict the masked tokens based on the context provided by the rest of the sequence. This approach encourages the model to learn representations that capture informative features of the protein sequences, enabling it to perform well on downstream tasks. ESM2 is trained on an enriched version of Uniref50^44^ dataset with over 60M sequences and comes in various sizes and training computational budgets. Notice that the standard Uniref50 dataset clusters sequences with at least 50% sequence identity and comprises approximately 45M sequences. We also investigate ESM-1b^29^ trained on the UniRef50 dataset as well as ESM-1v^38^ which is trained on the UniRef90^44^ dataset. The UniRef90 dataset clusters sequences with at least 90% sequence identity and contains about 138M sequences.

We further delve into the models from the ProtT5^30^ family. ProtT5 employs a variant of the Text-To-Text Transfer Transformer (T5) training procedure^24^ which utilizes an *encoder-decoder* architecture. ProtT5xl-bfd and ProtT5xxl-bfd models are trained on the BFD100 dataset. The BFD100 dataset is a clustered version of the Big Fantastic Database (BFD)^45,46^, where sequences with 100% identity are grouped, effectively removing duplicates. The dataset consolidates protein sequences from various sources and contains approximately 2.1B unique sequences. Subsequently, these models are trained on the UniRef50 dataset, resulting in the ProtT5xl and ProtT5xxl models. To provide an extensive outlook, we additionally examine ProtBert^30^, ProtAlbert^30^, ProtXLNet^30^, ProteinBert^39^ and Tasks Assessing Protein Embeddings (TAPE)^40^ models. ProtBert is trained on the UniRef100^44^ dataset, which consolidates 216M sequences from the UniProt Knowledgebase (UniProtKB)^47^, while ProtBert-bfd and ProteinBert on BFD100 and UniRef90, respectively. ProtAlbert and ProtXLNet utilize the BERT-based architectures ALBERT^48^ and XLNet^49^, respectively, and were trained on UniRef100. TAPE Bert^40^ is a BERT model, while TAPE Babbler^40^ is a unidirectional mLSTM model. Both TAPE models are trained on a dataset of approximately 32M protein sequences retrieved from the Pfam^50^ database. Finally, we consider SeqVec^41^, a model based on the bi-directional LSTM architecture of Embeddings from Language Models (ELMo)^51^, trained on the UniRef50 dataset.

### Embedding-based transfer learning

The first transfer learning approach, as shown in Figure 1a), begins by tokenizing the protein sequences using the model’s tokenizer. The tokenized sequence is then passed through the pre-trained PLM to generate token-level embeddings that capture both semantic and structural information. To create a fixed-size representation for each sequence, mean pooling is applied across the sequence length. These embeddings, along with their corresponding class labels, are then used to train a classifier to distinguish between antimicrobial and non-antimicrobial peptides (AMP classification). Mean pooling provides a straightforward mechanism to aggregate per-residue embeddings into a fixed-length vector, effectively preserving protein-level semantic information without introducing additional trainable parameters. This aggregation method has been widely adopted in protein representation tasks for its computational efficiency and robustness across varied sequence lengths, as demonstrated by Elnaggar et al.^30^ on multiple protein benchmarks.

For the AMP classification task, we utilize shallow classifiers such as Logistic Regression (LogReg), Support Vector Machines (SVMs), and Extreme Gradient Boosting (XGBoost). The incorporation of a diverse set of classifiers enables a thorough evaluation of how different machine learning models perform when leveraging PLM-generated embedding representations. For SVM and XGBoost classifiers, moderate hyper-parameter tuning is performed. The choice of shallow classifiers was primarily motivated by the relatively small size of the datasets in comparison to the embedding dimensions. Furthermore, the linear nature of the aggregated embedding space, empirically observed across various domains such as images and language, suggests that simple and shallow classifiers are capable of effectively capturing the underlying patterns and relationships within the data^22,52^.

### Efficient parameter fine-tuning

Adjusting the weights of a pre-trained model, rather than training task-specific classifiers harnessing its embeddings, is another transfer learning approach used in deep learning. Given sufficiently large sample size, it typically results in enhanced performance on downstream tasks^35,43,53^. This method involves the fine-tuning of the pre-trained model’s parameters towards the statistics of the new task while retaining its overall learned representations. For large models like PLMs and LLMs, full parameter fine-tuning can be computationally expensive, memory-intensive and may lead to overfitting. To address these issues, researchers have developed Parameter-Efficient Fine-Tuning (PEFT) techniques^54–56^. Among them, Low-Rank Adaptation (LoRA)^54^ and its variants^57–59^ have emerged as leading approaches due to their efficiency and simplicity. LoRA functions by introducing trainable low-rank matrices into specific layers of the pre-trained model (see also Figure 1b). These matrices serve as updates to the frozen model parameters, significantly reducing the number of trainable parameters during fine-tuning. Consequently, the low-rank nature of the updates, coupled with the preservation of the original model’s weights, significantly reduces the risks of catastrophic forgetting^60^ or overfitting which are common issues in many fine-tuning methods^61,62^. The utility of these PEFT methods for fine-tuning PLMs has been well-established in recent literature, with studies successfully applying them to diverse biological problems, demonstrating PEFT as a robust and efficient approach for specialized protein tasks^35,63–65^.

In this work, we deploy LoRA^54^ and its variant Quantized LoRA^57^ (QLoRA) for fine-tuning the PLMs to perform AMP classification. QLoRA introduces additional memory-saving innovations without compromising performance, including the 4-bit NormalFloat (NF4) data type for the weights, double quantization to further reduce memory usage, and paged optimizers to handle memory spikes. To extend the models towards the binary classification task (AMP vs non-AMP), a dense layer with tanh activation, dropout for regularization, and an output projection layer are attached to the final layer of the PLM’s encoder, serving as the classification head.

Moderate hyperparameter tuning was conducted to determine an optimal set of LoRA parameters, including the scaling factor, *α*, which adjusts the contribution of the low-rank updates, the rank, *r*, determining the size of the trainable matrices, and the dropout rate, used to regularize the low-rank updates. Additionally, typical training parameters such as learning rate, batch size, number of training epochs, and weight decay rate were optimized. Following the original LoRA methodology^54^ and standard practice in recent PLM studies^35,64^, we inserted the low-rank adapters evenly across all transformer layers of the PLMs, targeting the self-attention mechanism. Different combinations of query, key, value, and output projection matrices within the self-attention module were explored to identify the update configuration that demonstrates robust and consistent performance across all datasets and models under examination.

### Datasets description

This study evaluates the performance of PLMs on AMP classification using seven widely recognized datasets: XUAMP^6^, APD3^66^, CAMP^67,68^, dbAMP^69,70^, DRAMP^71,72^, LAMP^73,74^, and YADAMP^75^. These datasets vary considerably in size, ranging from as few as 406 sequences in CAMP to 3,072 sequences in XUAMP, underscoring the challenges posed by limited data availability in AMP classification. Each dataset has been carefully curated to balance the number of AMP and non-AMP sequences, with lengths ranging from 11 to 100 residues. Additionally, strict preprocessing ensures no sequences within the same subset share more than 40% sequence identity, measured using global alignment tools. Table 2 (also presented as Supplementary Table S1 for reference) presents a detailed summary of these datasets. Visualizations of the sequence embeddings for these datasets are provided in Supplementary Figures S1 and S2.

**Table 2:** Description of AMP datasets in terms of size, sequence length, mean/median sequence length, and average sequence-sequence identity.

Dataset	Number of Peptides	Length Range (residues)		Mean/Median Length		Sequence Identity (%)	
		AMP	Non-AMP	AMP	Non-AMP	AMP	Non-AMP
XUAMP	3072	16-100	28-100	62.9/63.0	77.1/79.0	25.4	28.7
DRAMP	2816	16-100	31-100	62.7/62.0	76.5/78.0	25.7	29.0
LAMP	2108	13-100	30-100	58.0/57.0	74.9/76.0	24.0	28.7
dbAMP	1044	17-100	32-100	52.5/48.0	75.5/77.0	23.9	28.8
APD3	988	13-100	31-100	48.1/43.0	75.6/77.0	24.1	28.9
YADAMP	648	11-100	33-100	31.4/32.0	77.1/79.0	25.3	29.0
CAMP	406	11-100	29-100	19.6/20.0	75.8/78.0	24.7	28.7

## Results

### Comparisons between PLMs: Scale matters

**Fig. 2:**
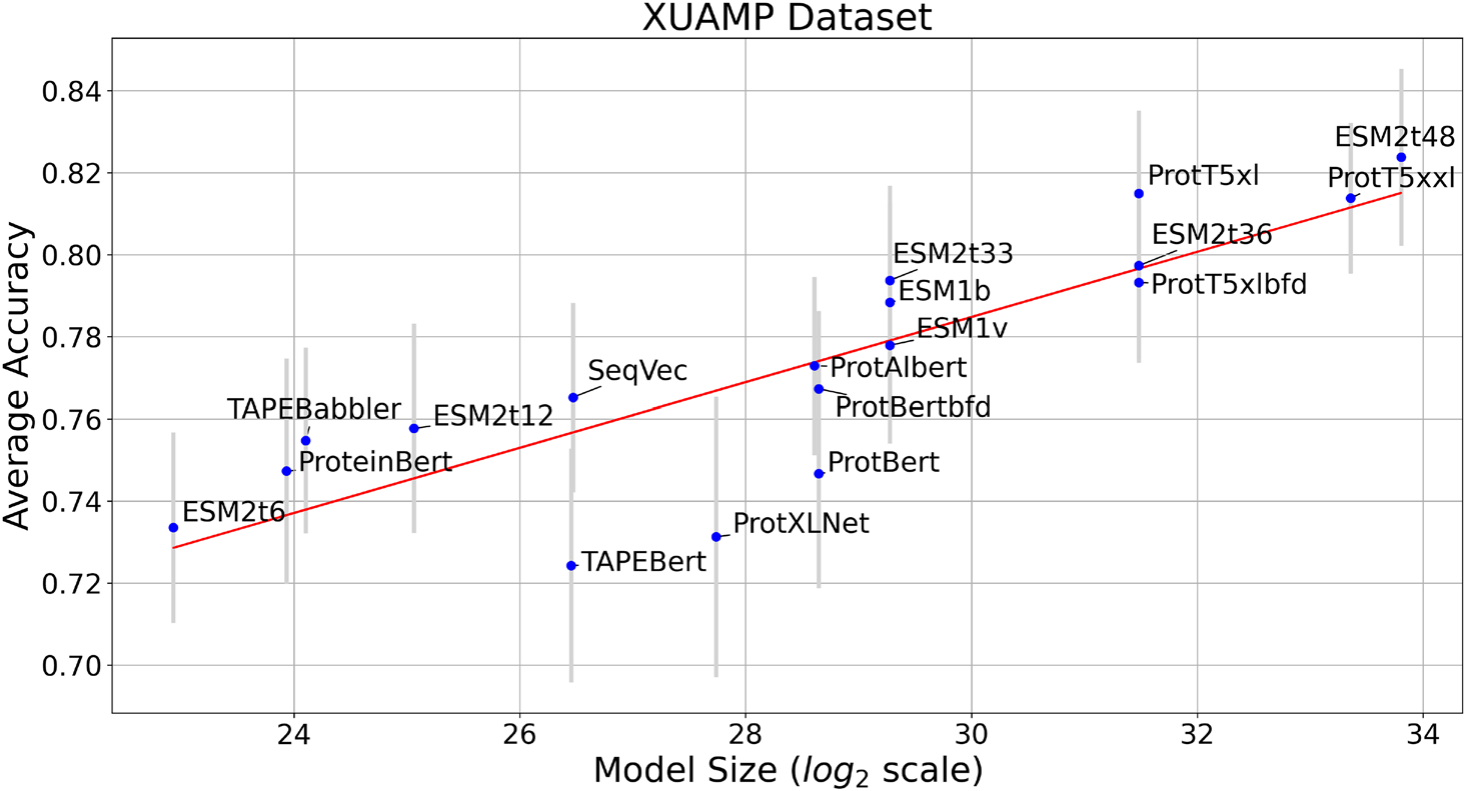
Average AMP accuracy plotted against model size on a logarithmic scale for the XUAMP dataset. Each point represents the mean accuracy, and the vertical grey lines indicate the respective standard deviation. The red line illustrates the linear fit described by the equation $y=0.008x+0.546$, which suggests that for every doubling of the model size, there is an associated 0.8% gain in accuracy.

This section compares the performance of PLMs on several AMP datasets using embedding-based transfer learning and shallow classifiers trained on the mean embedding vector generated by each PLM. Figure 2 showcases the relationship between average accuracy and model size in logarithmic scale for the XUAMP dataset. This evaluation employs three classifiers (LogReg, SVM and XGBoost), with moderate hyperparameter tuning performed for SVM and XGBoost (see Supplementary Tables S3-S4) to optimize their performance (more details in Supplementary section ‘Details on Training and Fine-Tuning’). The average accuracy and standard deviation are calculated by first performing 10-fold cross-validation for each classifier and then averaging the results across the three classifiers. It is evident from Fig. 2 that the model size plays a crucial role in improving the accuracy of the classifiers, indicating their elevated capacity to identify intricate patterns in protein sequences. As the PLM size increases, its proficiency in capturing and representing complex features of protein sequences improves, resulting in superior classification accuracy.

Apparently, the improvement scales relatively consistently with the logarithm of the model size. Indeed, the Spearman correlation between log-model size and AMP accuracy is 0.87, suggesting a strong relationship between the two quantities (see Supplementary Table S7 for per-dataset correlation coefficients). Moreover, the performance of the ESM2 family of models consistently exceeds the average trend (represented by the red line in Fig. 2), indicating that these models are particularly effective and achieve enhanced performance . This favorable behavior can be partially attributed to the high-quality training dataset that plays a significant role in enhancing the performance of the models. Similar trends are observed across all the tested AMP datasets, as shown in Supplementary Figures S4–S9, further reinforcing the consistency of our findings. The regression line statistics for all the datasets are summarized in Supplementary Table S8.

**Fig. 3:**
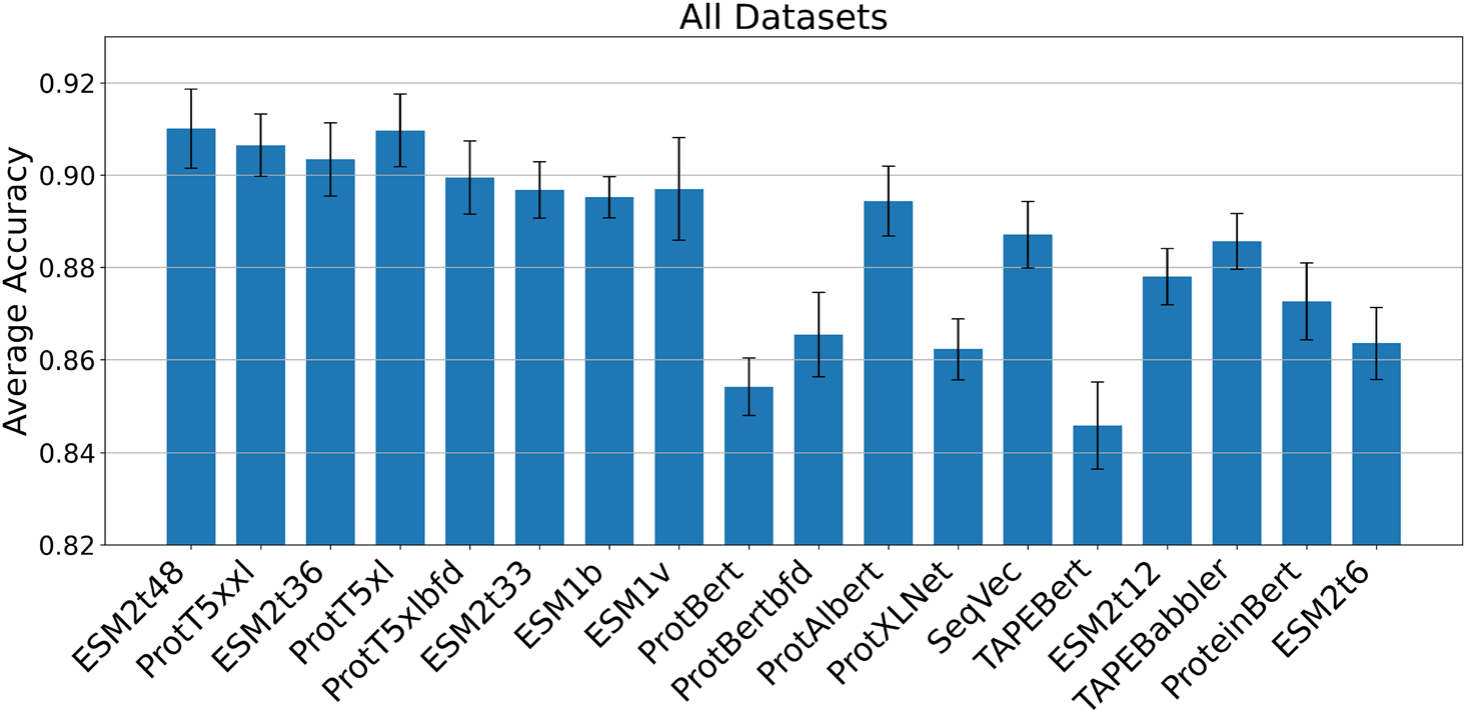
Average accuracy over all tested datasets with standard deviation using LogReg as a classifier. Models are arranged according to their size, from the largest (on the left) to the smallest (on the right).

Figure 3 generalizes the above observations via presenting the average accuracy over all AMP datasets using LogReg as a classifier (see Supplementary Figures S10–S11 for the other two classifiers). The ordering of the models in Fig. 3 is determined by their size, from the largest PLM to the smallest one. ESMt48 with 15B parameters and ProtT5xl with 3B parameters, exhibit the highest accuracy across all datasets. ProtT5xxl (11B) and ESM2t36 (3B) exhibit slightly lower accuracy, while certain BERT-based models, such as ProtBert (420M), TAPE Bert (92M), and ProtBert-bfd (240M), deliver even lower accuracy. The fact that ProtT5xl performs better than ProtT5xxl, which is almost 4 times larger, could be at least partially attributed to the computational budget constraints that were imposed for its training^30^, highlighting the significant role of additional training epochs in improving the performance of downstream tasks. Another observation from Fig. 3 is that ProtT5xl, which has been further trained with UniRef50, is significantly better from the ProtT5xl-bfd which is trained solely on the BFD dataset. This outcome aligns with the findings from Elnaggar et al.^30^ on different downstream tasks, suggesting that the use of a multi-stage training regimen, where a curated dataset like UniRef50 is used for a second training phase, can be beneficial for improved performance.

### Comparisons with Existing AMP Classifiers: Superior performance with minimal effort

Employing the same embedding-based transfer learning pipeline as previously, the top-performing PLMs are compared against state-of-the-art (SOTA) approaches in AMP classification, including sAMPpred-GAT^14^, amPEPpy^8^, AMPfun^9^ and AMPEP^11^. To ensure a fair comparison, we utilize the curated datasets from Yan et al.^14^ (summarized in Supplementary Table S2) and follow the same evaluation protocol. The methodology employed for dataset construction deviates from conventional evaluation practices such as *K*-fold cross validation, since special care is taken to avoid sequences in the training, validation, and test sets with large sequence identity. Consequently, these dataset splits also incorporate aspects of generalization error, and they actually correspond to a mixture of both classification and generalization error (we refer to Supplementary Figure S3, where those train and test sets are visualized using the UMAP algorithm^76^).

**Table 3:** Accuracy values across different AMP datasets for the train/test splits provided in the work of Yan *et al.*^14^. The last column corresponds to the average accuracy across all AMP datasets.

Model		Classifier	Dataset							Average
			XUAMP	DRAMP	LAMP	dbAMP	APD3	YADAMP	CAMP	
ESM2	t48	LogReg	**0.735**	0.766	0.884	**0.910**	0.922	0.968	0.978	0.880
		SVM	0.727	0.774	**0.888**	0.900	0.925	0.969	0.980	0.880
		XGBoost	0.727	0.764	0.853	0.885	0.898	0.961	**0.988**	0.868
	t36	LogReg	0.723	0.758	0.864	0.882	0.906	0.966	0.973	0.867
		SVM	0.725	0.761	0.868	0.884	0.909	0.958	0.973	0.868
		XGBoost	0.726	0.754	0.851	0.892	0.898	0.954	0.966	0.863
ProtT5	xxl	LogReg	0.723	0.752	0.871	0.899	0.912	0.957	0.970	0.869
		SVM	0.734	**0.779**	0.883	0.900	0.924	0.971	0.978	0.881
		XGBoost	0.724	0.762	0.873	0.908	0.917	**0.972**	0.975	0.876
	xl	LogReg	0.712	0.731	0.843	0.884	0.897	0.906	0.906	0.840
		SVM	**0.735**	0.762	0.864	0.895	0.907	0.944	0.936	0.863
		XGBoost	0.697	0.721	0.805	0.866	0.863	0.917	0.879	0.821
SOTA		sAMPpred-GAT	0.715	0.760	0.840	0.888	0.896	0.955	0.956	0.858
		amPEPpy	0.679	0.734	0.765	0.889	**0.939**	0.915	0.948	0.838
		AMPEP	0.661	0.712	0.755	0.766	0.936	0.969	0.973	0.825
		ADAM-HMM	0.684	0.736	0.872	0.886	0.886	0.927	0.869	0.837

Table 3 reports the accuracy of the four best-performing PLMs, compared to the results from Yan et al.^14^, which are derived by stand-alone deep learning models trained solely on AMP datasets. In six out of seven datasets, the embeddings from pretrained PLMs, when paired with shallow classifiers, outperformed the current SOTA stand-alone models. This emphasizes the benefits of embedding-based transfer learning approaches, suggesting that pretrained models can achieve superior performance in AMP classification with minimal effort. Moreover, the SVM classifier consistently produced higher accuracy values than other classifiers, although this difference was less pronounced for the ESM2 models compared to the ProtT5 models. Another key observation is that the largest model in each family consistently demonstrated the highest accuracy levels, indicating that these deeper models may reduce generalization error more effectively by providing more informative embeddings. Overall, we conclude that ESM2t48 and ProtT5xxl deliver the best performance in terms of AMP accuracy, a result that is further confirmed by other performance metrics reported in Supplementary Tables S9–S12.

### Parameter fine-tuning outperforms embedding-based transfer learning

Parameter fine-tuning, which allows the weights of the PLM to be updated, represents a more sophisticated transfer learning approach. In this section, we assess the accuracy of fine-tuned models on the AMP classification task and compare their performance to the embedding-based transfer learning approach. Specifically, we focus on LoRA and QLoRA, both of which are parameter-efficient fine-tuning techniques. Models from both ESM2 and ProtT5 families were selected for parameter fine-tuning due to their consistently high rankings in all previous experiments while the curated datasets and splits from Yan et al.^14^ were utilized for the evaluation.

**Fig. 4:**
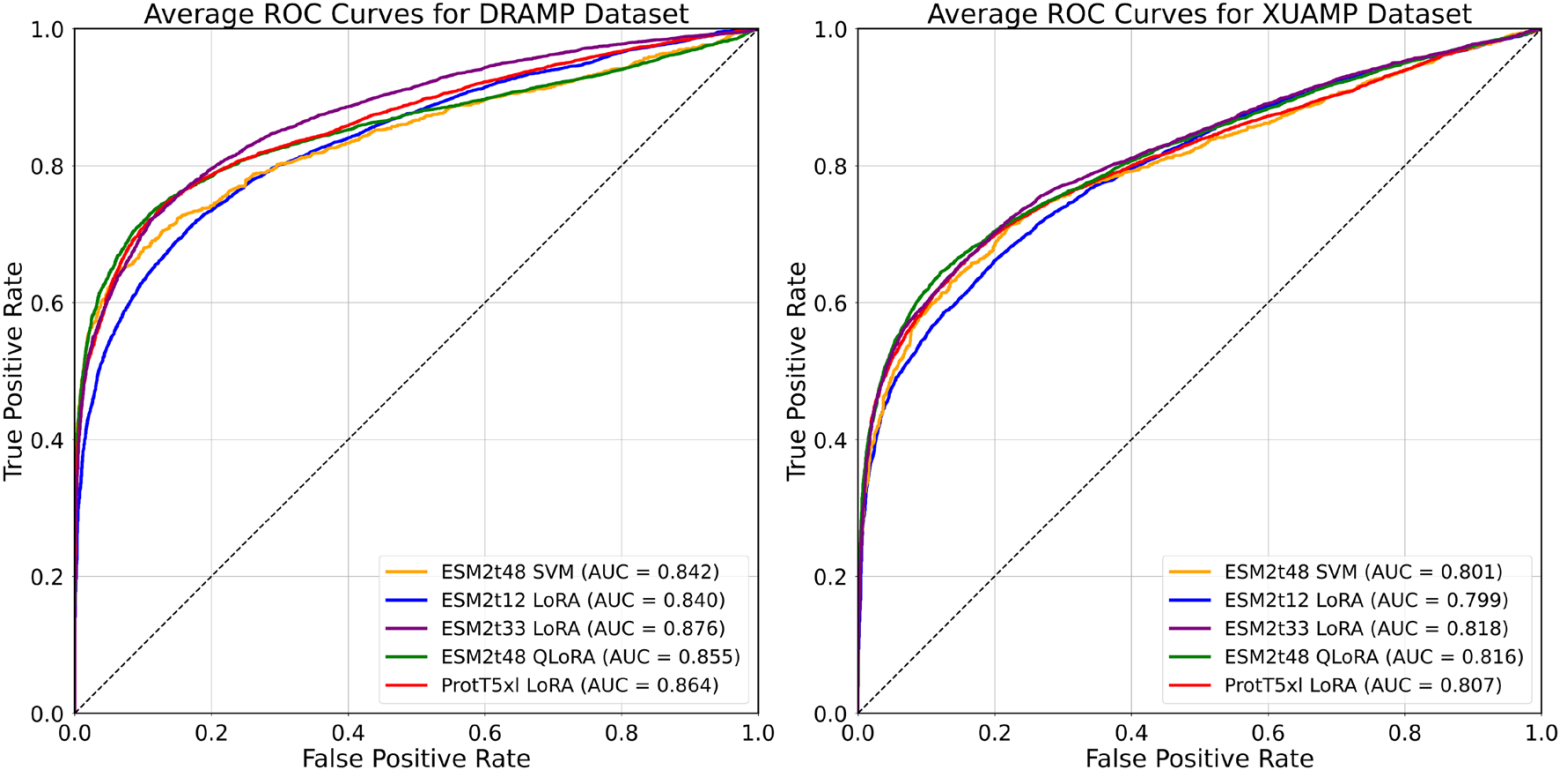
Average ROC curves for models evaluated on the DRAMP (left) and XUAMP (right) datasets. We compare the SVM classifier trained on the ESMt48 model’s embeddings (orange) with ESM2t12 (blue), ESM2t33 (purple), ESM2t48 (green) as well as ProtT5xl (red) fine-tuned with LoRA or QLoRA. The best-performing model from the embedding-based approach achieves performance comparable to the smallest fine-tuned ESM2 model. In contrast, all other PLMs fine-tuned with (Q)LoRA exhibit superior performance.

Experimentation with different combinations of target LoRA modules revealed that updating the query, key, and value matrices of the self-attention mechanism together produced the best results for the ESM2 family of models. For the ProtT5 models, we also updated the output weight matrix, which follows the self-attention mechanism. We set the rank $r=1$ for the weight update matrices, as this configuration not only yielded among the highest performance across all datasets but also introduces the fewest trainable parameters. We associate the low rank value to the limited number of samples available for fine-tuning, which is relatively small compared to the size of the PLM. Additional details on the hyperparameter values and tuning can be found in the ‘Details on Training and Fine-Tuning’ section and Tables S5-S6 in the Supplementary.

Figure 4 displays the receiver operating characteristic (ROC) curves for representative fine-tuned ESM2 and ProtT5 models on the DRAMP (left) and XUAMP (right) datasets. The results correspond to the average performance over five runs, each initialized with a different random seed. For comparison, the ROC curve of the SVM classifier, trained on the embeddings from the largest ESM2 model (ESM2t48), is also included. For the DRAMP dataset, the highest AUC value is achieved by fine-tuning ESM2t33 with LoRA, followed by ProtT5xl also fine-tuned with LoRA. Interestingly, the largest ESM2 model, fine-tuned with QLoRA, did not yield the highest AUC value, which may be attributed to the larger number of introduced trainable parameters leading to poor fitting, given the relatively small size of the training dataset. Furthermore, AUC values suggest that fine-tuning ESM2t12 offers classification performance comparable to the embedding-based transfer learning approach with the larger ESM2t48 model. ROC curve plots for the remaining datasets are available in the Supplementary Figure S12.

**Table 4:** Average AUC values for various fine-tuned PLMs across different datasets.

PLM		Transfer Learning	Dataset						
			XUAMP	DRAMP	LAMP	dbAMP	APD3	YADAMP	CAMP
ESM2	t48	SVM	0.801	0.842	0.962	0.972	0.977	0.996	**1.000**
	t12	LoRA	0.799	0.840	0.951	0.968	0.970	0.997	**1.000**
	t33	LoRA	**0.818**	0.877	0.960	0.976	0.977	0.997	**1.000**
	t33	QLoRA	0.817	**0.882**	0.961	0.977	0.974	0.997	**1.000**
	t36	LoRA	0.810	0.861	**0.965**	0.978	0.978	0.997	**1.000**
	t36	QLoRA	0.806	0.861	**0.965**	**0.980**	0.979	0.997	**1.000**
	t48	QLoRA	0.816	0.856	0.964	0.976	0.978	**0.998**	**1.000**
ProtT5	xxl	SVM	0.805	0.857	0.956	0.971	0.977	0.997	0.999
	xl	LoRA	0.807	0.864	0.962	0.972	0.980	0.997	0.999
	xxl	LoRA	0.802	0.852	0.963	0.975	0.978	0.996	**1.000**
SOTA		sAMPpred-GAT	0.777	0.827	0.917	0.952	0.954	0.994	**1.000**
		amPEPpy	0.742	0.759	0.855	0.940	0.972	0.968	0.978
		AMPEP	0.727	0.773	0.818	0.933	**0.983**	0.992	0.994
		ADAM-HMM	0.684	0.736	0.872	0.886	0.886	0.927	0.869
		AMPFUN	0.735	0.810	0.852	0.930	0.972	0.997	**1.000**

Table 4 reports a detailed comparison of average AUC values (five runs with different random seeds) for different PLMs fine-tuned with LoRA or QLoRA as well as stand-alone SOTA models. Standard deviations, being of the order of the third decimal, are omitted for clarity and are available in Supplementary Table S14. The results clearly demonstrate that fine-tuning consistently outperforms the embedding-based transfer learning approach in the AMP classification task, given the same model and dataset. The improvements observed in the DRAMP dataset, and to some extent in the XUAMP dataset, are particularly noteworthy. Furthermore, both LoRA and QLoRA show comparable performance in terms of AUC across all datasets, indicating that parameter quantization does not deteriorate performance. Apart from the AUC, the accuracy values of the fine-tuned PLMs have consistently improved, further demonstrating the enhanced predictive capabilities of these models. In particular, the highest accuracy achieved on the XUAMP dataset is 75.3% with the ESM2t36 model when fine-tuned using LoRA, while the highest accuracy on the DRAMP dataset reaches 79.4% with the same ESM2t36 model, but fine-tuned using QLoRA. For a detailed presentation of evaluation metrics, we refer to the Supplementary Tables S14–S19.

## Discussion

The unprecedented number of protein sequences available in public databases, coupled with the exponential growth of computational resources, presents a unique opportunity to deepen our knowledge of proteins, their structure and function through self-supervised learning. This work explores the transfer learning capabilities of various PLMs on the AMP classification task. The evaluation is conducted using real data from the literature and we aim to understand which characteristics of PLMs crucially contribute to efficiently capturing discriminative information between antimicrobial and non-antimicrobial peptide sequences. Our findings emphasize the *importance of scale* in achieving higher accuracy, with larger PLMs consistently outperforming their smaller counterparts. The results also highlight that PLMs can achieve SOTA results with minimal effort via transfer learning making AI-driven protein research more reliable and accessible to a broader audience. Indeed, the embedding-based transfer learning pipeline employed in this study demonstrates that highly effective AMP classification can be achieved without relying on complex architectures or extensive fine-tuning. This simplicity ensures that the embedding-based transfer learning framework can be readily adopted by researchers with minimal machine learning expertise. Additionally, fine-tuning methods such as LoRA and QLoRA further enhance PLM performance by adapting the models to specific datasets efficiently, while mitigating risks such as overfitting and catastrophic forgetting. This observation aligns well with recent studies on fine-tuning approaches of PLMs which have demonstrated notable performance gains across a range of classification or regression tasks^35^. Effectively, these parameter-efficient techniques balance computational demands and performance improvements, making them valuable tools for maximizing the utility of PLMs. We also investigated potential performance differences between the LoRA and QLoRA approaches via repeated experiments which were conducted across multiple datasets using the same training configurations. No statistically significant differences were observed in performance between LoRA and QLoRA as it is evident from Table 4 and Supplementary Tables S14–S19. In contrast, there are significant differences in computational requirements. QLoRA substantially reduces GPU memory usage requiring up to 9 times less memory compared to LoRA at the expense of roughly doubling the training time, while leaving inference time unaffected (see Supplementary Table S13).

Furthermore, we quantitatively assess the resilience of the two proposed pipelines under reduced data availability, evaluating their predictive power on progressively smaller subsets of the training data. When the training set size was halved, the performance drop was less than 1% for both the embedding-based and LoRA fine-tuning approaches. Reducing the sample size to one eighth resulted in decreases of around 3% and 4% for the two pipelines, respectively (see Supplementary Figures S13 and S14). In spite of showing slightly larger performance drops, the LoRA fine-tuned models consistently outperformed their embedding-based counterparts in terms of classification performance across all tested data sizes. In conclusion, both approaches maintained strong accuracy despite substantial data reduction, underscoring the robustness and lower risk for overfitting of transfer learning when applied to low-resource tasks such as AMP classification.

Although this study achieves SOTA performance in AMP classification, it likely represents a conservative estimate of PLMs’ potential. For instance, the use of mean pooling for embedding aggregation, while effective, may not fully exploit the rich information within the embeddings. Future research will explore more advanced aggregation techniques, such as task-specific attention mechanisms or bottleneck autoencoders, to enhance performance further. Moreover, the potential for improvement is supported by ongoing advancements in PLM architecture and training methodologies, which continue to produce models with greater representational power. For example, recent developments like xTrimoPGLM^33^ and ESM3^34^, featuring PLMs with 100 billion parameters, have shown superior performance compared to the largest ESM2 model across various downstream tasks. While the weights for these models are not yet publicly available, their success underscores the rapid progress in the field and the immense potential for further innovation to advance AMP classification and related applications.

## Supplementary Information

Supplementary Information.

## Data Availability

The datasets used in this study are publicly-available at: https://github.com/HongWuL/sAMPpred-GAT/tree/main/datasets.
