# Supplementary material for: Transfer learning on protein language models improves antimicrobial peptide classification
Source: Sci Rep. 2025 Oct 27;15:37456. doi: 10.1038/s41598-025-21223-y (PMC12559171; doi:10.1038/s41598-025-21223-y)
Supplement: Supplementary file 1 — Supplementary Information. [file 41598_2025_21223_MOESM1_ESM.pdf]

Supplementary Material for:
Transfer Learning on Protein Language Models Improves Antimicrobial Peptide Classification

Elias Georgoulis, Michaela Areti Zervou and Yannis Pantazis

Datasets Overview

In this study, we utilize Antimicrobial Peptide (AMP) datasets to evaluate the performance of various Protein Language Models (PLMs) from Xu *et al.*[1] and Yan *et al.*[2]. Specifically, the datasets from Xu et al. are employed independently in the *"Comparisons between PLMs: Scale Matters"* section. For the *"Comparisons with Existing AMP Classifiers: Superior Performance with Minimal Effort"* and *"Parameter Fine-Tuning Outperforms Embedding-Based Transfer Learning"* sections, we use the curated datasets and splits from Yan *et al.*[2]. The datasets from Yan *et al.* are carefully designed to minimize sequence identity between training, validation, and test sets, ensuring unbiased and robust evaluations.

Xu *et al.* curated datasets

Table S1 summarizes the seven AMP datasets from Xu *et al.*[1], utilized for the comparison of the Protein Language Models (PLMs) under examination, detailing the number of peptides, the range of sequence lengths, the mean/median lengths for both AMP and non-AMP classes, and the average pairwise sequence identity. Each dataset contains the same number of AMP and non-AMP sequences. These datasets are publicly available at the following link:
https://github.com/HongWuL/sAMPpred-GAT/tree/main/datasets/independent%20test%20datasets.

The sequence identity percentage in Tables S1 and S2 represents the average pairwise identity of sequences within each class for a given dataset. Identity scores between pairs of sequences are calculated using Biopython's function `pairwise2.align.globalxx` for global alignment. This alignment implies that only matches between both sequences are counted. The score for each pair is normalized dividing it by the max length of the two sequences and expressed as a percentage. The overall average is derived by computing the mean of these percentages for all pairs within the subset.

Table S1. Summary of datasets from Xu *et al.*

Dataset	Number of Peptides	Length Range (residues)		Mean/Median Length		Sequence Identity (%)	
		AMP	Non-AMP	AMP	Non-AMP	AMP	Non-AMP
**XUAMP**	3072	16-100	28-100	62.9/63.0	77.1/79.0	25.4	28.7
**DRAMP**	2816	16-100	31-100	62.7/62.0	76.5/78.0	25.7	29.0
**LAMP**	2108	13-100	30-100	58.0/57.0	74.9/76.0	24.0	28.7
**dbAMP**	1044	17-100	32-100	52.5/48.0	75.5/77.0	23.9	28.8
**APD3**	988	13-100	31-100	48.1/43.0	75.6/77.0	24.1	28.9
**YADAMP**	648	11-100	33-100	31.4/32.0	77.1/79.0	25.3	29.0
**CAMP**	406	11-100	29-100	19.6/20.0	75.8/78.0	24.7	28.7

UMAP plots

Figures S1 and S2 display the Uniform Manifold Approximation and Projection (UMAP)[3] plots for the embedding representations generated by different models for each dataset listed in Table S1. The visualizations across the AMP datasets clearly show that the larger ESM2t48 model forms clusters that are more distinctly separated between the two classes compared to the smaller ESM2t6 model. This distinction is expected to translate directly into performance improvements.

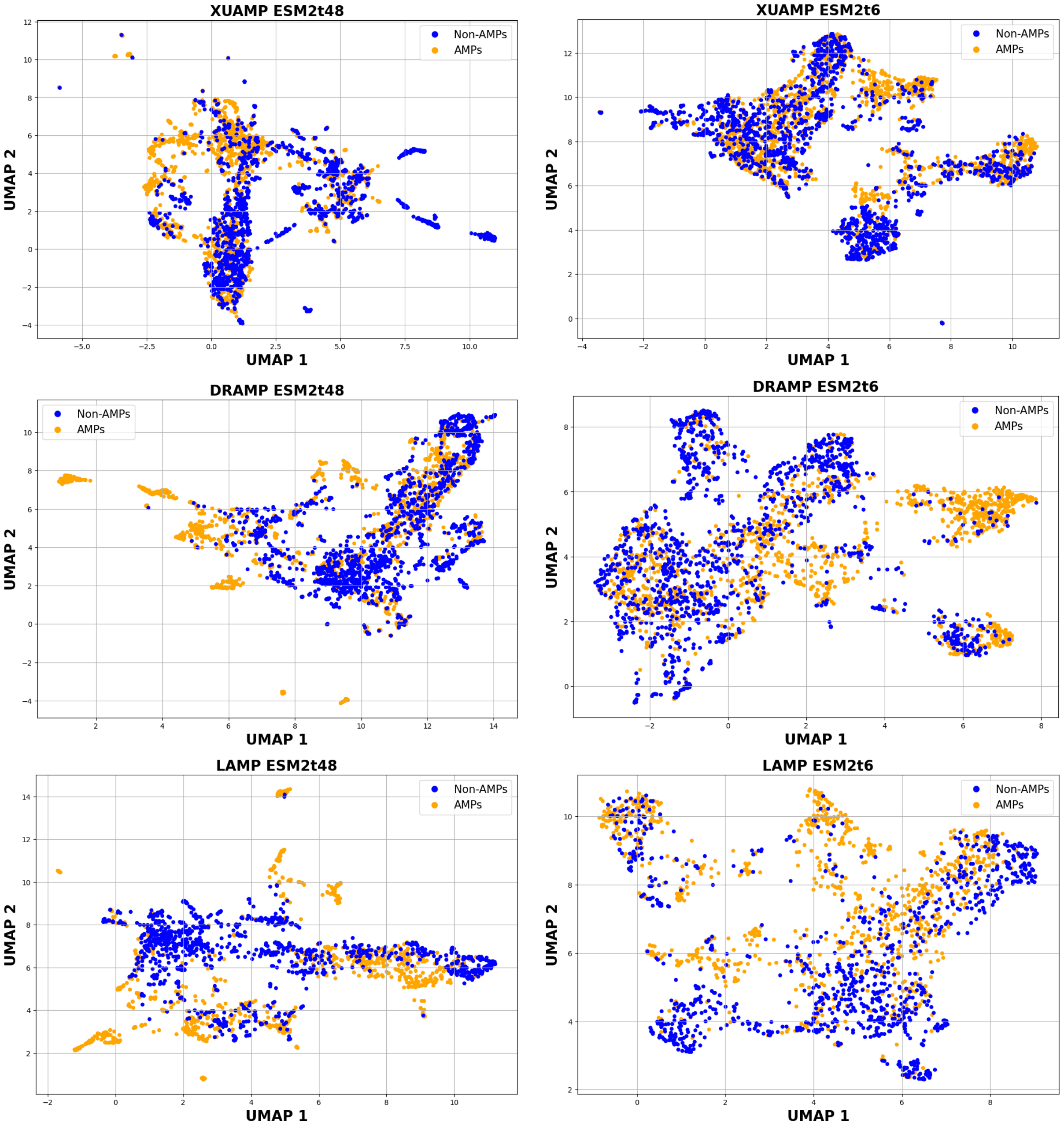

Figure S1. UMAP visualization of embeddings derived from ESM2t48 (left) and ESM2t6 (right) models for XUAMP, DRAMP, and LAMP datasets.

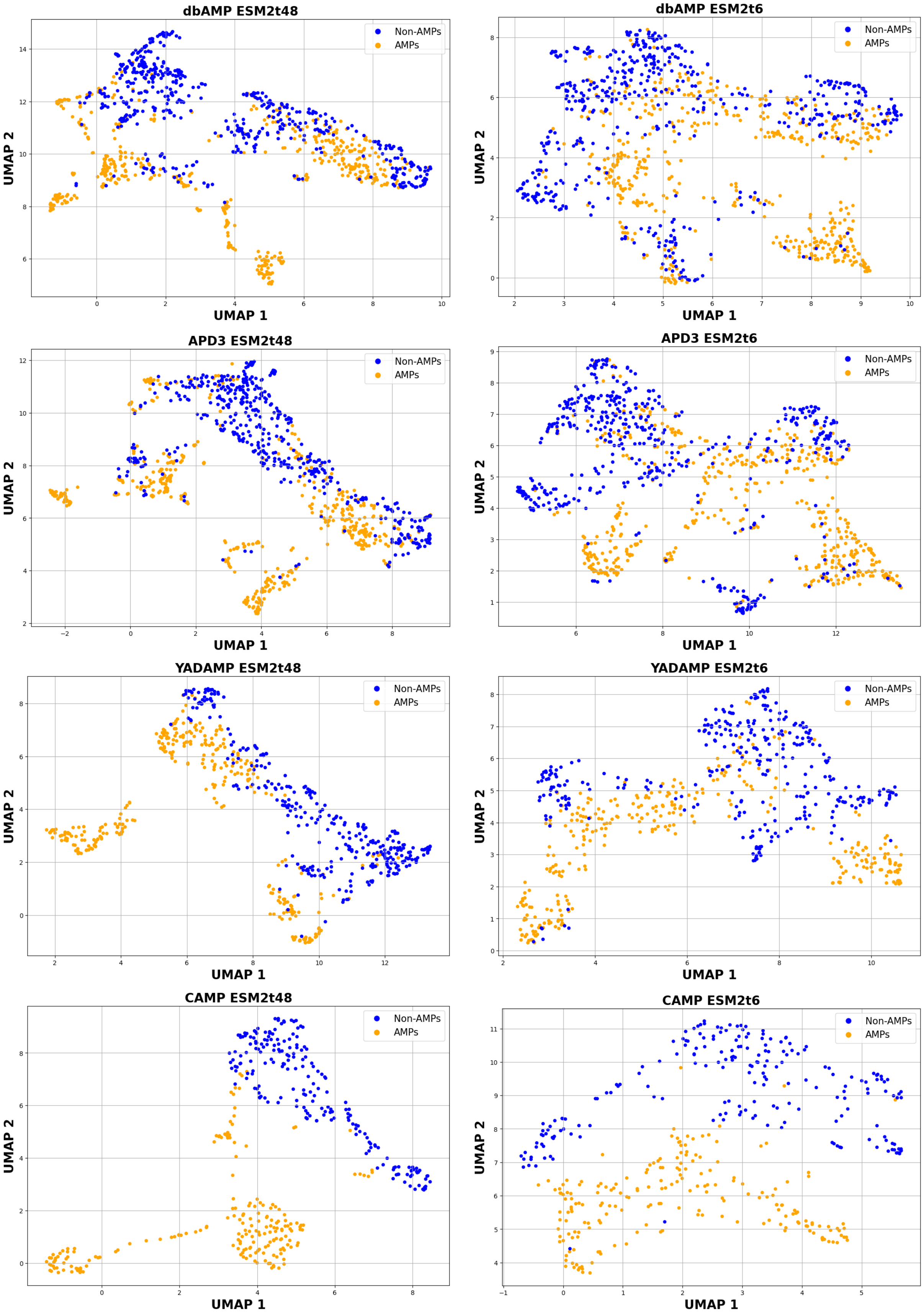

Figure S2. UMAP visualization of embeddings derived from ESM2t48 (left) and ESM2t6 (right) models for dbAMP, APD3, YADAMP, and CAMP datasets.

Yan *et al.* curated datasets

To ensure a fair comparison with the other state-of-the-art methods, we utilize the curated datasets and their corresponding splits from Yan *et al.*[2] Specifically, for the training of our classifiers we use the datasets that can be found at:
https://github.com/HongWuL/sAMPpred-GAT/tree/main/datasets/train%20datasets.

As training datasets we utilize the datasets annotated as "NAME_pretrain_train_negative.fasta" for the non-AMPs and "NAME_pretrain_train_positive.fasta" for AMPs. These datasets are summarized in Table S2. As validation datasets for the fine-tuning of our classifiers we use the datasets annotated as "NAME_pretrain_val_negative(or positive).fasta". The test datasets correspond to those from *Xu et al.*, previously described in Table S1.

Table S2. Summary of datasets from Yan et al, detailing dataset size, sequence length range, mean/-median sequence lengths, and average pairwise sequence identity.

Dataset	Number of Peptides		Length Range (residues)		Mean/Median Length		Sequence Identity (%)	
	AMP	Non-AMP	AMP	Non-AMP	AMP	Non-AMP	AMP	Non-AMP
**XUAMP**	4426	4431	11-100	28-100	47.2/43.0	76.7/79.0	22.4	29.1
**LAMP**	3858	3852	11-100	28-100	45.8/41.0	76.7/79.0	22.3	29.1
**DRAMP**	3976	3632	11-100	28-100	45.0/38.0	76.7/79.0	22.3	29.2
**dbAMP**	4174	4116	11-100	28-100	46.5/41.0	76.7/79.0	23.3	29.1
**APD3**	4189	4162	11-100	28-100	47.4/43.0	76.8/79.0	22.5	29.2
**YADAMP**	4345	4244	11-100	28-100	47.5/43.0	76.6/79.0	22.4	29.1
**CAMP**	4394	4324	11-100	28-100	47.4/43.0	76.7/79.0	22.5	29.2

UMAP plots

Figure S3 displays the UMAP visualizations of embedding representations generated by different models for the XUAMP, DRAMP, and LAMP datasets. In these visualizations, the training datasets (summarized in Table S2) are represented by dark blue points for non-AMPs and dark orange points for AMPs, while the test datasets (summarized in Table S1) are depicted in light blue for non-AMPs and light orange for AMPs.

The UMAP plots in Figure S3 demonstrate a noticeable distinction between clusters representing the training and test set samples of the same class, reflecting the low sequence identity between these sets as enforced during dataset design.

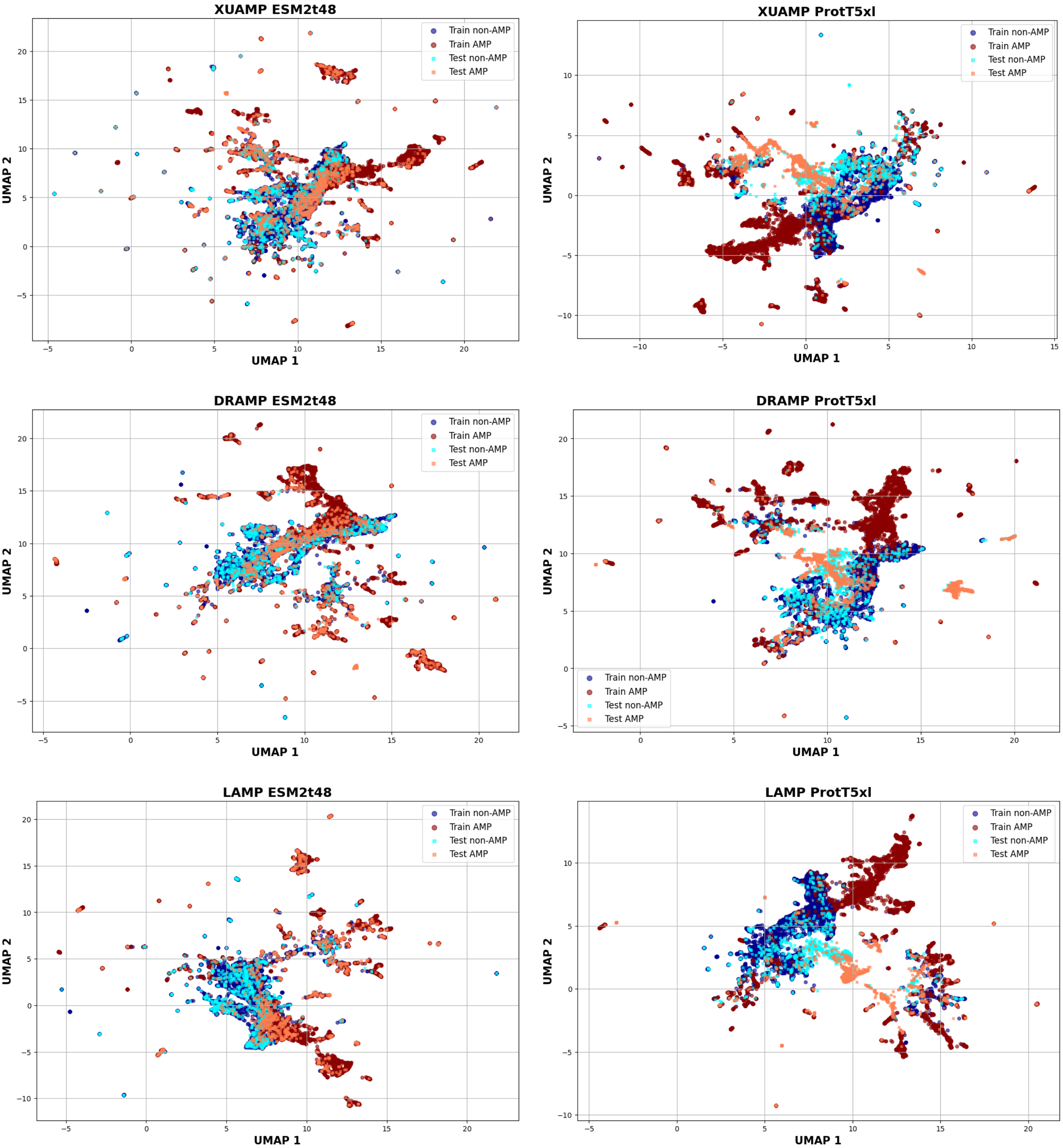

Figure S3. UMAP visualizations of embeddings generated by the ESM2t48 (left) and ProtT5xl (right) models for datasets listed in Table S2 (training sets) and Table S1 (test sets).

Details on Training and Fine-Tuning

Comparisons between PLMs: Scale matters

Tables S3 and S4 present the hyper-parameters examined for Support Vector Machines (SVMs) and eXtreme Gradient Boosting (XGBoost), respectively. A grid search combined with 10-fold cross-validation was performed independently for each Protein Language Model (PLM) and dataset to identify the best configurations.

In the case of SVMs, a second, more targeted grid search experiment was performed, focusing on values around the best-performing regularization parameter $C = 1$. This search identified $C = 1.8$ as the optimal value across PLMs and datasets. The combination of Radial Basis Function (RBF) kernel and `scale` gamma option consistently delivered the highest performance.

For XGBoost we opted on using the default configuration as it showed consistent performance across different PLMs and datasets.

For the Logistic Regression classifier we use the default scikit-learn configuration, increasing only the maximum number of training iterations (`max_iter`) to 1000 to ensure convergence.

Table S3. Hyperparameters and their values explored for SVM tuning

Hyperparameter	Values Tested
Kernel Type	Linear, RBF, Polynomial
C Value	0.01, 0.1, 1, 10, 100, 1000
Degree (for Polynomial)	2, 3, 4
Gamma	Scale, Auto, 0.01, 0.1

Table S4. Hyperparameters and their values explored for XGBoost tuning

Hyperparameter	Values Tested
n_estimators	50, 100, 150, 200, 250, 500
learning_rate	0.01, 0.1, 0.2
max_depth	3, 4, 5, 6, 7, 10
gamma	0, 0.1, 0.2, 1
subsample	0.7, 0.8, 0.9, 1
colsample_bytree	0.7, 0.8, 0.9, 1
reg_alpha	0, 0.1, 0.5, 1

Comparisons with Existing AMP Classifiers: Superior performance with minimal effort

The optimal hyperparameter configuration for each dataset and model in this section was selected based on performance on the corresponding validation set (see Section 'Yan *et al.* Curated Datasets'). The hyperparameters evaluated for SVMs and XGBoost are summarized in Tables S3 and S4.

For SVMs, the most frequently observed optimal configuration consisted of $C = 10$, the Radial Basis Function (RBF) kernel, and the `scale` option for gamma.

For XGBoost, the most common optimal configuration consisted of $\texttt{max\_depth} = 6$, $\texttt{learning\_rate} = 0.1$, $\texttt{n\_estimators} = 150$, $\texttt{subsample} = 0.8$, and $\texttt{colsample\_bytree} = 0.8$.

For the Logistic Regression classifier we use the default scikit-learn configuration, increasing only the maximum number of training iterations (`max_iter`) to 1000 to ensure convergence.

Parameter Fine-Tuning Outperforms Embeddings-Based Transfer Learning

For Low Rank Adaptation (LoRA)[4] fine-tuning, we utilize the PEFT library (Parameter-Efficient Fine-Tuning) in conjunction with the Hugging Face Transformers' Trainer.

For ESM2 models, we employ the `EsmForSequenceClassification` class from the Hugging Face Transformers library, which integrates a classification head tailored for sequence classification tasks. This implementation utilizes the CLS token generated by the ESM2 model as input to the classification head. For ProtT5 models, we leverage the implementation by Schmirler *et al.*[5]. This approach applies mean pooling to the per-amino-acid embeddings generated by the ProtT5 model, using the resulting per-protein embeddings as input to the classification head.

Table S5 summarizes the LoRA-specific hyperparameter values and configurations explored during fine-tuning. Table S6 provides the general training configuration options tested, which are not specific to LoRA.

The datasets utilized for these experiments are described in 'Yan et al. curated datasets' section. Throughout the fine-tuning process, model performance is assessed at the end of each epoch using the validation set. The model instance achieving the highest F1 score on the validation set is selected as the final model. In the last step the model's performance is evaluated on the test set.

Table S5. Range of values for the LoRA specific hyper-parameters

**LoRA Parameter**	**Value**
**r (Rank)**	1, 2, 4, 8
**LoRA Alpha**	0.1, 1, 2, 4, 8
**Bias Configuration**	'lora_only', 'none'
**LoRA Dropout**	0, 0.1, 0.2, 0.3
**Target Modules**	query, key, value, output projection

Table S6. Range of values for the remaining training hyper-parameters

**Configuration Option**	**Value**
**Optimizer**	AdamW
**Learning Rate**	0.01, 0.005, 0.001, 0.0001
**Weight Decay**	0, 0.01, 0.1
**Learning Rate Scheduler**	Linear, Cosine
**Batch Size (Train)**	1, 4, 8, 16, 32, 64
**Number of Epochs**	3, 5, 10
**Gradient accumulation steps**	1, 2, 4

Lower values for the rank, r, yielded better performance, prompting us to select $r = 1$ to additionally minimize the number of additional trainable parameters. We hypothesize that the effectiveness of low r values is driven by the limited number of available samples for fine-tuning, which constrains the model's capacity to benefit from additional parameters.

The configuration choices that yielded the most consistent results across the various models and datasets are detailed below.

LoRA Hyperparameters:

- **Rank (r):** 1
- **LoRA scaling factor (α):** 1
- **Bias Configuration:** `lora_only`
- **LoRA Dropout:** 0.1
- **Target Modules:** `query`, `key`, `value`

Training Hyperparameters:

- **Batch Size:** 16
- **Number of Epochs:** 3 (ESM2 models), 5 (ProtT5 models)
- **Learning Rate:** 0.001
- **Learning Rate Scheduler:** `Linear`
- **Weight Decay:** 0.1

The ESM2t48 model was fine-tuned using QLoRA[6] to address computational constraints arising from the model's large size, while all other configuration choices remained unchanged.

For the ProtT5 models the inclusion of the output projection matrix of the attention mechanism as a target module for LoRA, led to better performance, so we included it for the fine-tuning of these models.

For the ProtT5xxl model, gradient accumulation with a step size of 2 was employed to effectively increase the batch size from 8 to 16, addressing computational constraints. Additionally, the learning rate was increased to 0.002.

Additional Results

Comparisons between PLMs: Scale matters

Figures S4-S9 depict the average accuracy and standard deviation across all classifiers under examination (LogReg, SVM, and XGBoost), utilizing embedding representations derived from different PLMs. The average accuracy and standard deviation are calculated by first performing 10-fold stratified cross-validation for each classifier and then averaging the results across the three classifiers. The figures correspond to the following datasets: DRAMP, LAMP, dbAMP, YADAMP, APD3, and CAMP. The horizontal axis displays the model size on a logarithmic scale, and the red line represents the fitted regression line.

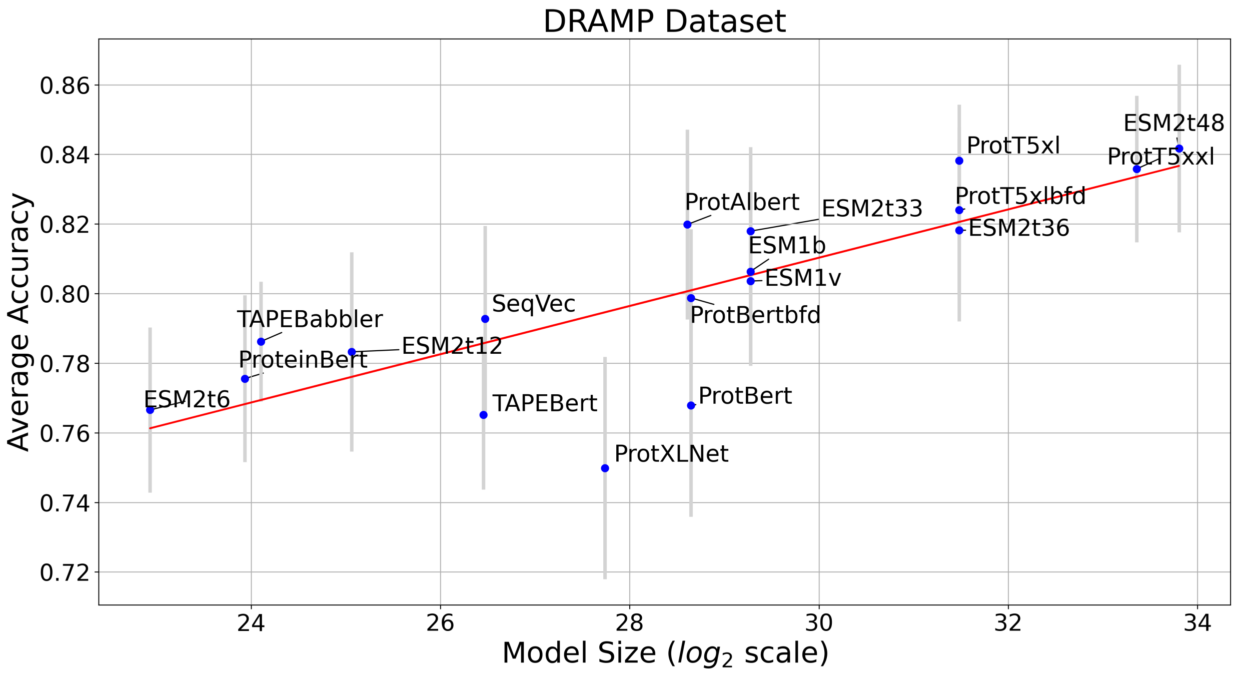

Figure S4. Average accuracy over model size on the DRAMP Dataset. $y = 0.007x + 0.602$. Spearman correlation coefficient: 0.832 P-value: 2×10^{-5}. Pearson correlation coefficient: 0.793 P-value: 9×10^{-5}.

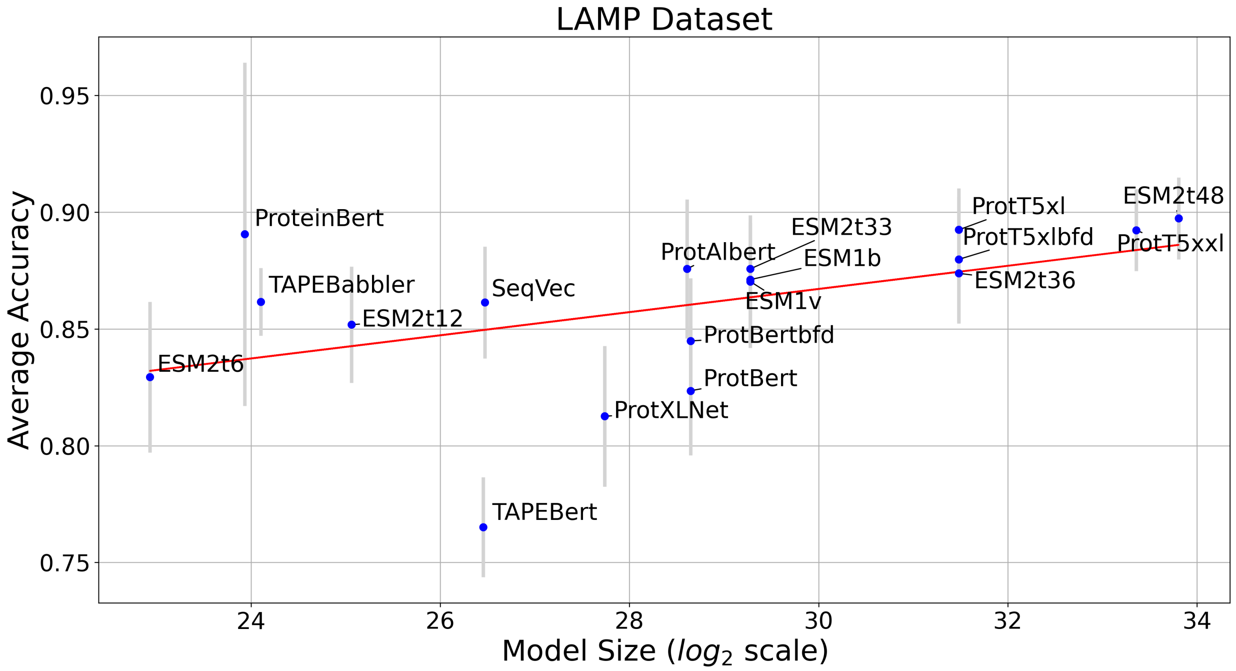

Figure S5. Average accuracy over model size on the LAMP Dataset. $y = 0.005x + 0.718$. Spearman correlation coefficient: 0.615 P-value: 0.007. Pearson correlation coefficient: 0.466 P-value: 0.051.

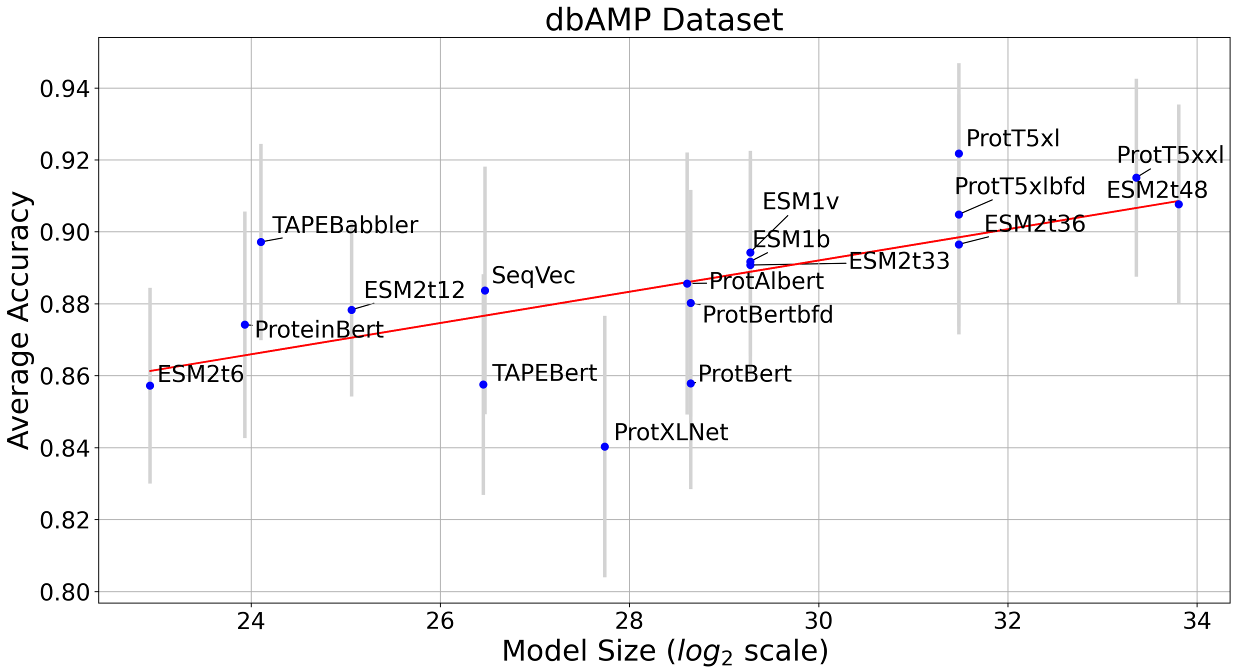

Figure S6. Average accuracy over model size on the dbAMP Dataset. $y = 0.004x + 0.762$. Spearman correlation coefficient: 0.753 P-value: 3×10^{-4}. Pearson correlation coefficient: 0.636 P-value: 0.005.

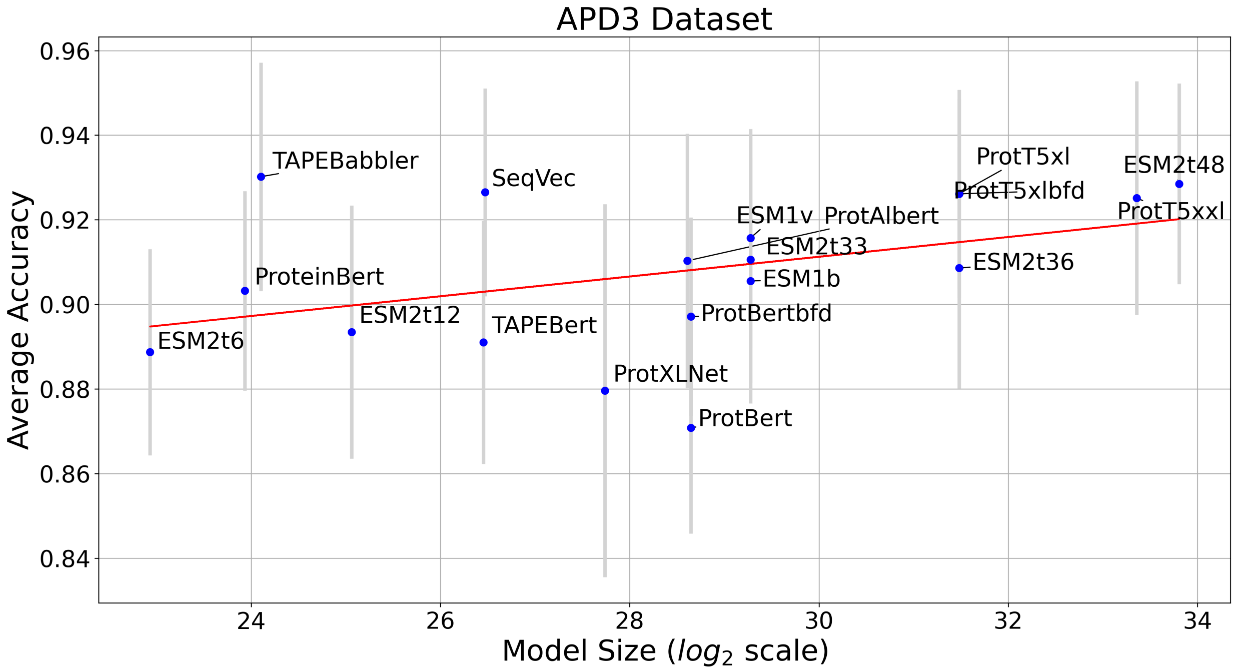

Figure S7. Average accuracy over model size on the APD3 Dataset. $y = 0.002x + 0.841$. Spearman correlation coefficient: 0.440 P-value: 0.068. Pearson correlation coefficient: 0.414 P-value: 0.088.

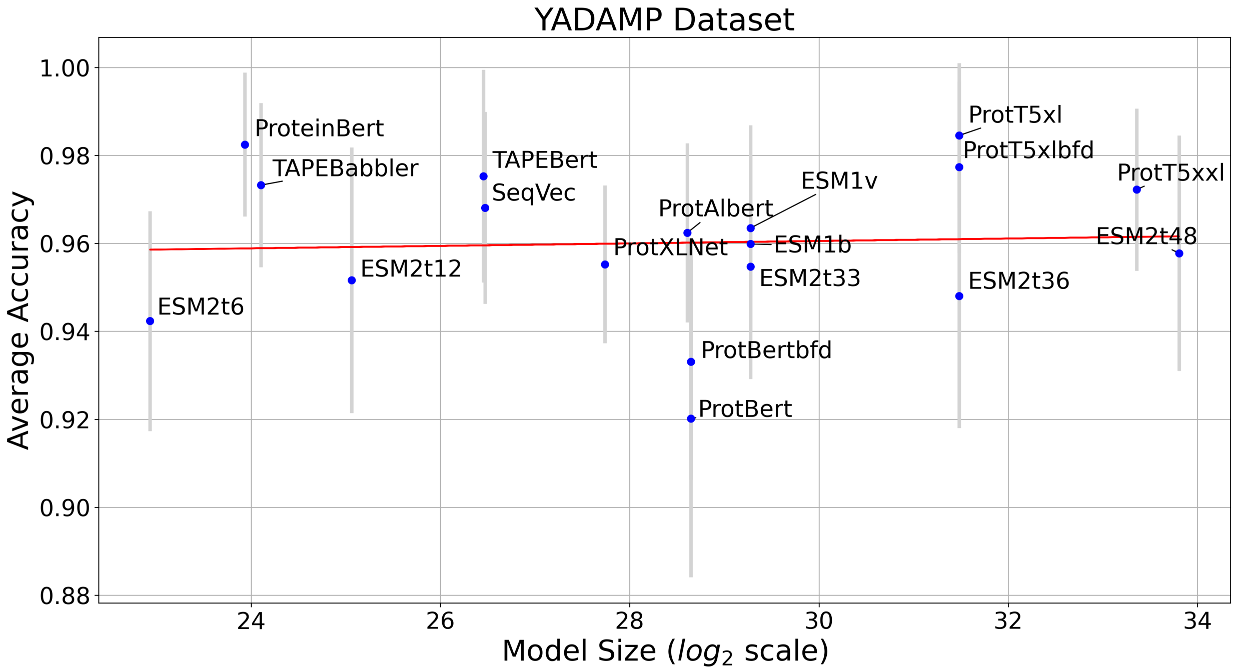

Figure S8. Average accuracy over model size on the YADAMP Dataset. $y = 3 \times 10^{-4}x + 0.952$. Spearman correlation coefficient: 0.054 P-value: 0.832. Pearson correlation coefficient: 0.052 P-value: 0.838.

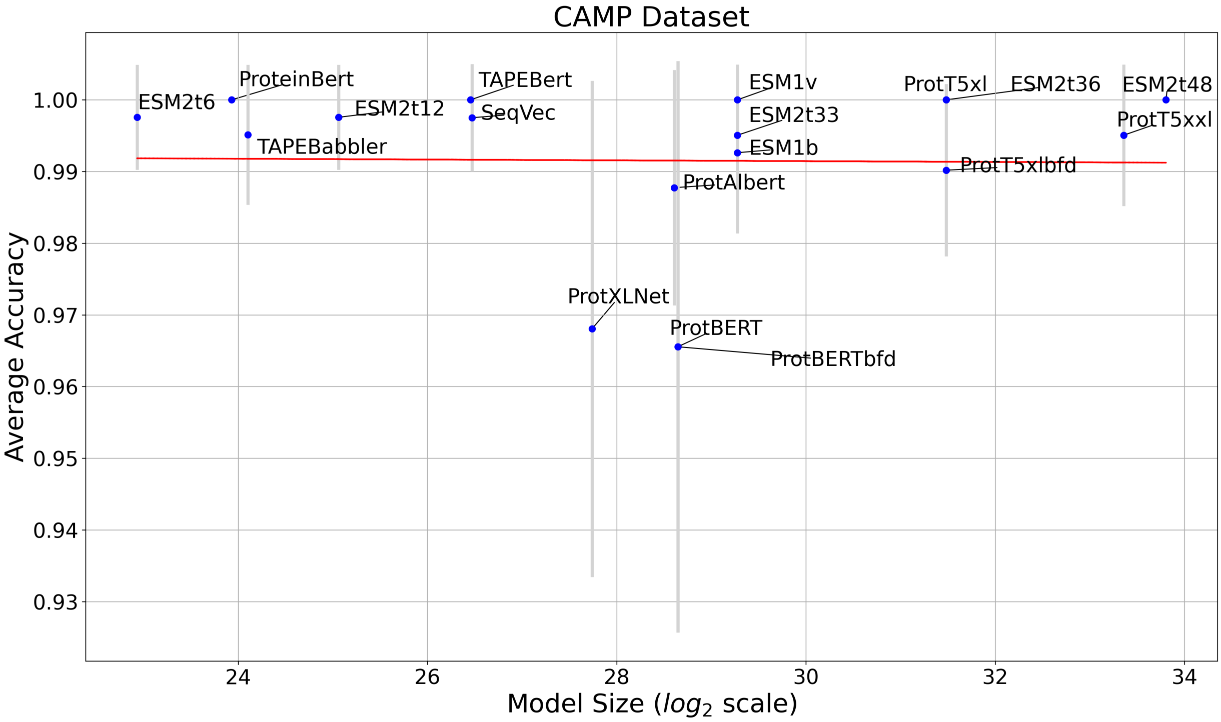

Figure S9. Average accuracy over model size on the CAMP Dataset. $y = -9 \times 10^{-5}x + 0.988$. Spearman correlation coefficient: -0.022 P-value: 0.932. Pearson correlation coefficient: -0.017 P-value: 0.947.

The detailed statistical results, including the correlation coefficients and their respective P-values, are summarized in Table S7. Additionally, Table S8 consolidates the regression line statistics for each dataset, reporting the average accuracy, its standard deviation as well as the slope and the intercept of the linear fit.

The results reveal a clear trend: datasets with lower baseline accuracy, indicative of greater complexity, exhibit steeper slopes in their regression lines compared to those with higher baseline accuracy. This pattern suggests that model size increases have a more pronounced effect on improving accuracy in more challenging datasets. Larger models are likely more capable at learning nuanced features of protein sequences, potentially enabling improved generalization and downstream performance.

Table S7. Correlation Results on Various Datasets

**Dataset**	**Spearman**		**Pearson**	
	**Coefficient**	**P-value**	**Coefficient**	**P-value**
**XUAMP**	0.871	3×10^{-6}	0.842	10^{-5}
**DRAMP**	0.832	2×10^{-5}	0.793	9×10^{-5}
**LAMP**	0.615	0.007	0.466	0.051
**dbAMP**	0.753	3×10^{-4}	0.636	0.005
**APD3**	0.440	0.068	0.414	0.088
**YADAMP**	0.054	0.832	0.052	0.838
**CAMP**	-0.022	0.932	-0.017	0.947

Table S8. Regression Line Statistics

Dataset	Avg. Accuracy	Std. Dev.	Slope	Intercept
**XUAMP**	0.772	0.038	0.008	0.546
**DRAMP**	0.800	0.037	0.007	0.602
**LAMP**	0.859	0.044	0.005	0.718
**dbAMP**	0.885	0.036	0.004	0.762
**APD3**	0.908	0.033	0.002	0.841
**YADAMP**	0.960	0.029	3×10^{-4}	0.952
**CAMP**	0.985	0.028	-9×10^{-5}	0.988

Figures S10 and S11 display histograms that illustrate the average accuracy of the SVM and XGBoost classifiers across all examined datasets, respectively. In each histogram, the models are ordered sequentially by their size, from the largest to the smallest.

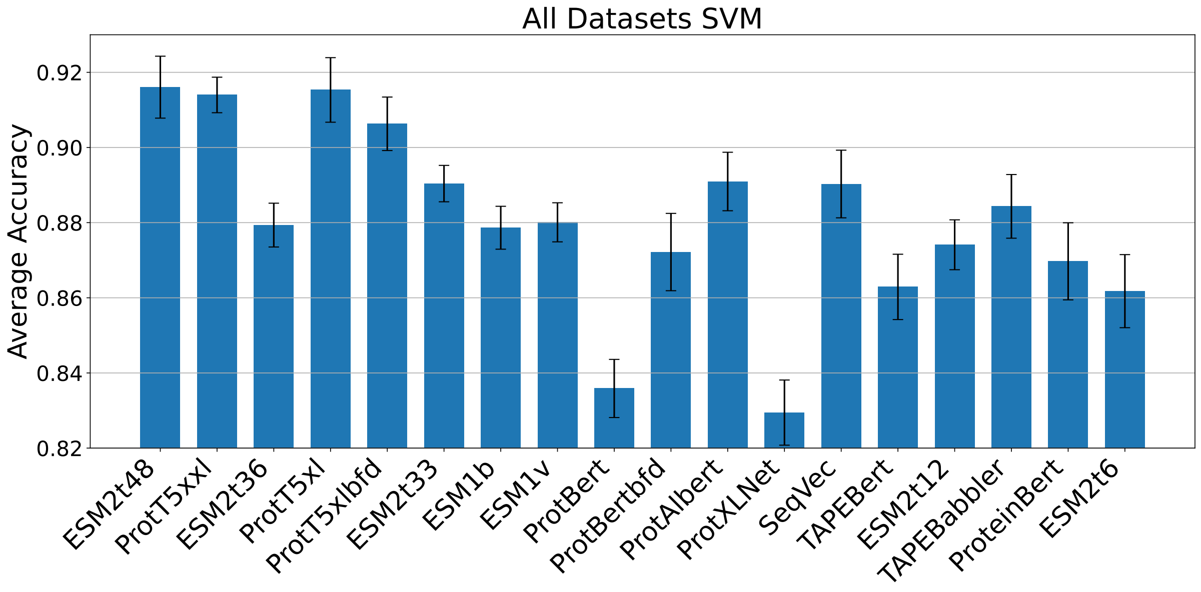

Figure S10. Average classification accuracy and standard deviation of the SVM classifier across all datasets, using embedding-based transfer learning from various PLMs, ordered by model size.

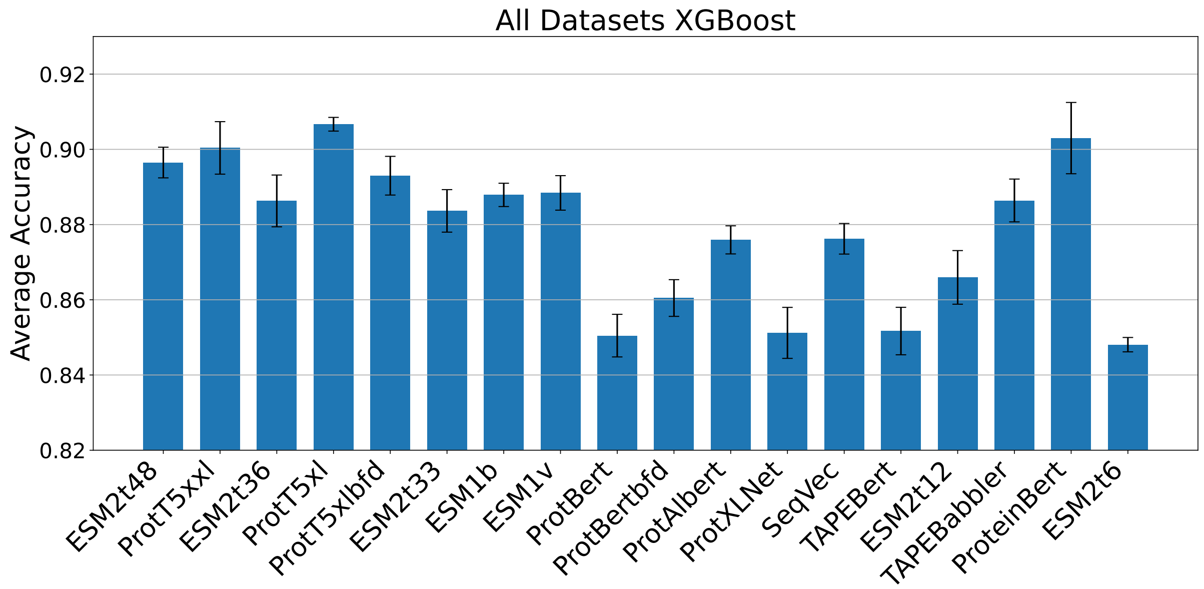

Figure S11. Average classification accuracy and standard deviation of the XGBoost classifier across all datasets, using embedding-based transfer learning from various PLMs, ordered by model size.

Comparisons with Existing AMP Classifiers: Superior performance with minimal effort

Tables S9 through S12 present AUC, Precision, Recall and F1 score values for LogReg, SVM and XGBoost classifiers trained on embedding representations derived from different PLMs, for all examined datasets.

We observe low Recall values for the XUAMP, DRAMP, and LAMP datasets, which subsequently translate to lower accuracy, F1 score and AUC metrics for these datasets.

Table S9. AUC values for various PLMs and classifiers across AMP datasets.

Model	Classifier	Dataset						
		**XUAMP**	**DRAMP**	**LAMP**	**dbAMP**	**APD3**	**YADAMP**	**CAMP**
**ESM2 t48**	LogReg	**0.811**	0.853	**0.962**	**0.973**	0.976	0.996	**1.000**
	SVM	0.801	0.842	**0.962**	0.972	**0.977**	0.996	**1.000**
	XGBoost	0.786	0.840	0.955	0.965	0.969	0.994	**1.000**
**ESM2 t36**	LogReg	0.788	0.833	0.955	0.966	0.969	0.995	**1.000**
	SVM	0.789	0.822	0.951	0.966	0.971	0.993	**1.000**
	XGBoost	0.776	0.830	0.952	0.966	0.967	0.991	0.998
**ProtT5 xxl**	LogReg	0.785	0.838	0.954	0.971	0.972	0.995	**1.000**
	SVM	0.805	**0.857**	0.956	0.971	**0.977**	**0.997**	0.999
	XGBoost	0.785	0.841	0.957	0.968	0.973	**0.997**	**1.000**
**ProtT5 xl**	LogReg	0.765	0.814	0.932	0.954	0.961	0.967	0.975
	SVM	0.792	0.828	0.937	0.955	0.962	0.979	0.974
	XGBoost	0.773	0.815	0.917	0.945	0.944	0.964	0.955

Table S10. Precision values for various PLMs and classifiers across AMP datasets.

Model	Classifier	Dataset						
		XUAMP	DRAMP	LAMP	dbAMP	APD3	YADAMP	CAMP
**ESM2 t48**	LogReg	0.896	0.939	**0.969**	0.959	0.958	0.955	0.958
	SVM	0.890	0.949	0.966	0.960	0.955	0.955	0.962
	XGBoost	0.890	0.942	0.956	0.953	0.946	0.949	**0.976**
**ESM2 t36**	LogReg	0.873	0.932	0.949	0.944	0.947	0.952	0.949
	SVM	0.880	0.924	0.947	0.945	0.949	0.941	0.949
	XGBoost	0.886	0.933	0.960	0.957	0.950	0.951	0.944
**ProtT5 xxl**	LogReg	0.850	0.914	0.946	0.941	0.932	0.938	0.944
	SVM	**0.899**	**0.950**	0.963	0.948	0.958	**0.964**	0.958
	XGBoost	0.889	0.942	0.961	**0.969**	0.946	0.964	0.953
**ProtT5 xl**	LogReg	0.848	0.882	0.912	0.937	0.919	0.934	0.902
	SVM	0.843	0.839	0.898	0.899	0.887	0.900	0.886
	XGBoost	0.847	0.880	0.900	0.930	**0.913**	0.914	0.889

Table S11. Recall values for various PLMs and classifiers across AMP datasets.

Model	Classifier	Dataset						
		XUAMP	DRAMP	LAMP	dbAMP	APD3	YADAMP	CAMP
**ESM2 t48**	LogReg	0.533	0.568	0.793	0.856	0.883	0.981	**1.000**
	SVM	0.518	0.579	0.804	0.835	0.893	0.985	**1.000**
	XGBoost	0.518	0.563	0.740	0.810	0.844	0.975	**1.000**
**ESM2 t36**	LogReg	0.523	0.557	0.770	0.812	0.860	0.981	**1.000**
	SVM	0.521	0.568	0.780	0.816	0.864	0.978	**1.000**
	XGBoost	0.519	0.548	0.732	0.820	0.840	0.957	0.990
**ProtT5 xxl**	LogReg	0.542	0.556	0.787	0.852	0.889	0.978	**1.000**
	SVM	0.527	0.589	0.797	0.847	0.887	0.978	**1.000**
	XGBoost	0.512	0.558	0.777	0.843	0.885	0.981	**1.000**
**ProtT5 xl**	LogReg	0.516	0.534	0.760	0.824	0.870	0.873	0.911
	SVM	**0.577**	**0.649**	**0.823**	**0.889**	**0.933**	**1.000**	**1.000**
	XGBoost	0.480	0.512	0.685	0.791	0.804	0.920	0.867

Table S12. F1 score values for various PLMs and classifiers across AMP datasets.

Model	Classifier	Dataset						
		XUAMP	DRAMP	LAMP	dbAMP	APD3	YADAMP	CAMP
**ESM2 t48**	LogReg	0.668	0.708	0.872	**0.905**	0.919	0.968	0.978
	SVM	0.655	0.719	**0.877**	0.893	**0.923**	0.970	0.981
	XGBoost	0.655	0.705	0.834	0.876	0.892	0.962	**0.988**
**ESM2 t36**	LogReg	0.654	0.697	0.850	0.873	0.901	0.967	0.974
	SVM	0.655	0.704	0.855	0.876	0.905	0.959	0.974
	XGBoost	0.654	0.690	0.831	0.883	0.892	0.954	0.966
**ProtT5 xxl**	LogReg	0.662	0.691	0.859	0.894	0.910	0.958	0.971
	SVM	0.665	0.727	0.872	0.895	0.921	0.971	0.978
	XGBoost	0.650	0.701	0.859	0.902	0.914	**0.972**	0.976
**ProtT5 xl**	LogReg	0.642	0.665	0.829	0.877	0.894	0.903	0.907
	SVM	**0.685**	**0.732**	0.858	0.894	0.909	0.947	0.940
	XGBoost	0.613	0.648	0.778	0.855	0.855	0.917	0.878

Parameter Fine-Tuning Outperforms Embedding-Based Transfer Learning

Tables S14 through S19 present the average metric values for AUC, Accuracy, Precision, Recall, F1 score and MCC across five independent runs of each model's fine-tuning. Each run is initialized with a different random seed to ensure robustness and account for variability in model performance. All models were fine-tuned using LoRA, with the exception of ESM2t48, which was fine-tuned using QLoRA due to computational resource constraints.

To evaluate potential performance differences between models fine-tuned with LoRA and those fine-tuned with QLoRA under the same training configurations, additional experiments were conducted using QLoRA across different sizes of ESM2 models. No statistically significant differences observed compared to models fine-tuned with LoRA. These findings indicate that the QLoRA approach does not introduce notable changes in performance metrics, suggesting comparable efficacy to conventional LoRA fine-tuning.

Table S13 reports the mean per-epoch training time and peak GPU memory for LoRA and QLoRA across different ESM2 and ProtT5 models of varying sizes, all fine-tuned for five epochs. QLoRA reduces peak memory usage by up to nine-fold (ESM2 t36), at the expense of roughly doubling per-epoch training time. As model size grows, the memory savings become more significant, with a corresponding increase in execution time overhead, underscoring the compute-for-memory trade-off of QLoRA.

Table S13. Per-epoch training time and peak GPU memory for LoRA vs QLoRA fine-tuning over five epochs (mean $\pm$ standard deviation). All experiments were conducted on a single NVIDIA A100-SXM4-80GB GPU.

Model	LoRA		QLoRA	
	Time (s)	Memory (GB)	Time (s)	Memory (GB)
ESM2 t6	14.96 ± 0.40	0.33 ± 0.00	25.54 ± 0.42	0.13 ± 0.00
ESM2 t12	23.26 ± 0.09	0.89 ± 0.00	46.77 ± 0.05	0.21 ± 0.00
ESM2 t30	58.31 ± 3.45	2.89 ± 0.06	117.82 ± 0.70	0.39 ± 0.00
ESM2 t33	64.00 ± 1.30	7.54 ± 0.00	132.15 ± 2.81	0.96 ± 0.00
ESM2 t36	119.13 ± 0.94	24.16 ± 0.00	240.42 ± 1.38	2.80 ± 0.02
ESM2 t48	-	-	818.20 ± 3.18	13.97 ± 1.61
ProtT5 xl	48.25 ± 1.34	8.59 ± 0.87	68.66 ± 0.29	3.62 ± 0.39
ProtT5 xxl	48.67 ± 1.03	34.28 ± 3.52	73.71 ± 3.96	14.54 ± 1.23

Table S14. AUC values (mean $\pm$ standard deviation) for different fine-tuned PLMs across different AMP datasets.

PLM		Fine-Tuning Method	Dataset						
			XUAMP	DRAMP	LAMP	dbAMP	APD3	YADAMP	CAMP
ESM2	t12	LoRA	0.799 (0.002)	0.840 (0.002)	0.951 (0.000)	0.968 (0.001)	0.970 (0.004)	0.997 (0.000)	**1.000 (0.000)**
	t12	QLoRA	0.800 (0.002)	0.840 (0.001)	0.951 (0.001)	0.965 (0.001)	0.971 (0.002)	0.997 (0.000)	**1.000 (0.000)**
	t33	LoRA	**0.818 (0.001)**	0.877 (0.004)	0.960 (0.001)	0.976 (0.000)	0.977 (0.001)	0.997 (0.000)	**1.000 (0.000)**
	t33	QLoRA	0.817 (0.003)	**0.882 (0.001)**	0.961 (0.001)	0.977 (0.001)	0.974 (0.002)	0.997 (0.001)	**1.000 (0.000)**
	t36	LoRA	0.810 (0.002)	0.861 (0.002)	**0.965 (0.001)**	0.978 (0.001)	0.978 (0.001)	0.997 (0.001)	**1.000 (0.000)**
	t36	QLoRA	0.806 (0.003)	0.861 (0.004)	**0.965 (0.001)**	**0.980 (0.001)**	0.979 (0.001)	0.997 (0.000)	**1.000 (0.000)**
	t48	QLoRA	0.816 (0.004)	0.856 (0.006)	0.964 (0.001)	0.976 (0.002)	0.978 (0.000)	**0.998 (0.000)**	**1.000 (0.000)**
ProtT5	xl	LoRA	0.807 (0.002)	0.864 (0.009)	0.962 (0.001)	0.972 (0.002)	**0.980 (0.001)**	0.997 (0.001)	0.999 (0.001)
	xxl	LoRA	0.802 (0.002)	0.852 (0.014)	0.963 (0.001)	0.975 (0.002)	0.978 (0.002)	0.996 (0.001)	**1.000 (0.000)**

Table S15. Accuracy values (mean ± standard deviation) for different fine-tuned PLMs across different AMP datasets.

PLM		Fine-Tuning Method	Dataset						
			XUAMP	DRAMP	LAMP	dbAMP	APD3	YADAMP	CAMP
ESM2	**t12**	LoRA	0.723 (0.002)	0.750 (0.001)	0.880 (0.002)	0.902 (0.003)	0.909 (0.004)	0.962 (0.002)	0.972 (0.004)
	**t12**	QLoRA	0.716 (0.002)	0.752 (0.003)	0.878 (0.002)	0.900 (0.003)	0.917 (0.003)	0.964 (0.003)	0.991 (0.004)
	**t33**	LoRA	0.748 (0.004)	0.771 (0.002)	0.891 (0.004)	0.920 (0.004)	0.925 (0.004)	0.972 (0.003)	0.978 (0.006)
	**t33**	QLoRA	0.748 (0.002)	0.780 (0.003)	0.896 (0.002)	0.919 (0.004)	0.924 (0.005)	0.975 (0.003)	0.982 (0.002)
	**t36**	LoRA	**0.753 (0.002)**	0.777 (0.004)	0.897 (0.005)	0.921 (0.004)	**0.934 (0.001)**	0.972 (0.003)	0.981 (0.002)
	**t36**	QLoRA	0.744 (0.003)	**0.794 (0.003)**	0.898 (0.004)	0.917 (0.006)	0.934 (0.003)	**0.979 (0.003)**	**0.985 (0.002)**
	**t48**	QLoRA	0.744 (0.009)	0.778 (0.005)	**0.909 (0.002)**	**0.923 (0.011)**	0.930 (0.005)	0.973 (0.003)	0.984 (0.004)
ProtT5	**xl**	LoRA	0.746 (0.006)	0.789 (0.004)	0.909 (0.004)	0.917 (0.005)	0.928 (0.004)	0.978 (0.003)	0.980 (0.003)
	**xxl**	LoRA	0.743 (0.004)	0.784 (0.004)	0.907 (0.004)	0.920 (0.004)	0.934 (0.002)	0.978 (0.004)	0.985 (0.004)

Table S16. Precision values (mean ± standard deviation) for different fine-tuned PLMs across different AMP datasets.

PLM		Fine-Tuning Method	Dataset						
			XUAMP	DRAMP	LAMP	dbAMP	APD3	YADAMP	CAMP
ESM2	**t12**	LoRA	0.858 (0.005)	0.905 (0.003)	0.930 (0.004)	0.951 (0.004)	0.937 (0.004)	0.940 (0.005)	0.947 (0.007)
	**t12**	QLoRA	0.853 (0.002)	0.913 (0.002)	0.923 (0.002)	0.905 (0.004)	0.942 (0.023)	0.950 (0.004)	**0.982 (0.007)**
	**t33**	LoRA	0.890 (0.002)	0.937 (0.003)	0.960 (0.006)	0.968 (0.002)	0.955 (0.003)	0.971 (0.005)	0.958 (0.011)
	**t33**	QLoRA	0.890 (0.003)	0.931 (0.003)	0.951 (0.003)	0.964 (0.005)	0.954 (0.005)	0.967 (0.003)	0.965 (0.004)
	**t36**	LoRA	0.897 (0.005)	0.950 (0.005)	0.964 (0.004)	0.974 (0.002)	**0.969 (0.004)**	0.958 (0.013)	0.964 (0.004)
	**t36**	QLoRA	0.887 (0.005)	0.918 (0.013)	0.961 (0.003)	0.972 (0.005)	0.966 (0.003)	0.972 (0.004)	0.970 (0.004)
	**t48**	QLoRA	**0.910 (0.005)**	**0.956 (0.009)**	**0.971 (0.002)**	**0.977 (0.006)**	0.965 (0.006)	0.961 (0.004)	0.969 (0.007)
ProtT5	**xl**	LoRA	0.868 (0.012)	0.919 (0.011)	0.943 (0.006)	0.962 (0.011)	0.946 (0.005)	0.967 (0.003)	0.961 (0.006)
	**xxl**	LoRA	0.892 (0.013)	0.923 (0.016)	0.951 (0.008)	0.962 (0.009)	0.950 (0.005)	**0.975 (0.004)**	0.970 (0.008)

Table S17. Recall values (mean ± standard deviation) for different fine-tuned PLMs across different AMP datasets.

PLM		Fine-Tuning Method	Dataset						
			XUAMP	DRAMP	LAMP	dbAMP	APD3	YADAMP	CAMP
ESM2	**t12**	LoRA	0.534 (0.003)	0.559 (0.002)	0.823 (0.005)	0.848 (0.004)	0.877 (0.011)	0.988 (0.003)	**1.000 (0.000)**
	**t12**	QLoRA	0.521 (0.003)	0.556 (0.006)	0.825 (0.004)	0.893 (0.003)	0.891 (0.020)	0.980 (0.003)	**1.000 (0.000)**
	**t33**	LoRA	0.565 (0.007)	0.580 (0.004)	0.817 (0.013)	0.870 (0.009)	0.893 (0.007)	0.973 (0.009)	**1.000 (0.000)**
	**t33**	QLoRA	0.566 (0.004)	0.606 (0.006)	0.835 (0.004)	0.871 (0.006)	0.891 (0.007)	0.983 (0.008)	**1.000 (0.000)**
	**t36**	LoRA	0.572 (0.004)	0.584 (0.012)	0.824 (0.012)	0.865 (0.008)	0.896 (0.004)	0.987 (0.012)	**1.000 (0.000)**
	**t36**	QLoRA	0.559 (0.006)	**0.646 (0.016)**	0.830 (0.009)	0.859 (0.015)	0.899 (0.005)	0.986 (0.003)	**1.000 (0.000)**
	**t48**	QLoRA	0.540 (0.017)	0.584 (0.017)	0.844 (0.003)	0.867 (0.028)	0.891 (0.007)	0.987 (0.005)	**1.000 (0.000)**
ProtT5	**xl**	LoRA	**0.580 (0.013)**	0.634 (0.013)	**0.871 (0.009)**	0.869 (0.010)	0.908 (0.009)	**0.990 (0.004)**	**1.000 (0.000)**
	**xxl**	LoRA	0.554 (0.003)	0.621 (0.010)	0.857 (0.016)	**0.874 (0.011)**	**0.916 (0.006)**	0.980 (0.006)	**1.000 (0.000)**

Table S18. F1 score values (mean ± standard deviation) for different fine-tuned PLMs across different AMP datasets.

PLM		Fine-Tuning Method	Dataset						
			XUAMP	DRAMP	LAMP	dbAMP	APD3	YADAMP	CAMP
ESM2	t12	LoRA	0.659 (0.003)	0.691 (0.001)	0.873 (0.003)	0.897 (0.004)	0.906 (0.005)	0.963 (0.002)	0.973 (0.004)
	t12	QLoRA	0.647 (0.003)	0.691 (0.005)	0.871 (0.002)	0.899 (0.003)	0.915 (0.002)	0.964 (0.003)	**0.991 (0.004)**
	t33	LoRA	0.691 (0.006)	0.717 (0.003)	0.883 (0.006)	0.916 (0.005)	0.923 (0.004)	0.972 (0.003)	0.978 (0.006)
	t33	QLoRA	0.692 (0.003)	0.734 (0.004)	0.889 (0.003)	0.915 (0.004)	0.921 (0.005)	0.975 (0.003)	0.982 (0.002)
	t36	LoRA	**0.699 (0.003)**	0.723 (0.008)	0.888 (0.007)	0.916 (0.005)	0.931 (0.001)	0.972 (0.002)	0.982 (0.002)
	t36	QLoRA	0.686 (0.004)	**0.758 (0.007)**	0.891 (0.005)	0.912 (0.007)	0.931 (0.004)	**0.979 (0.003)**	0.985 (0.002)
	t48	QLoRA	0.678 (0.014)	0.725 (0.010)	0.903 (0.002)	**0.918 (0.013)**	0.927 (0.005)	0.974 (0.003)	0.984 (0.004)
ProtT5	xl	LoRA	0.695 (0.009)	0.750 (0.007)	**0.905 (0.004)**	0.913 (0.005)	0.927 (0.004)	**0.979 (0.003)**	0.980 (0.003)
	xxl	LoRA	0.683 (0.004)	0.742 (0.005)	0.902 (0.006)	0.916 (0.005)	**0.933 (0.002)**	0.978 (0.004)	0.985 (0.004)

Table S19. MCC values (mean ± standard deviation) for different fine-tuned PLMs across different AMP datasets.

PLM		Fine-Tuning Method	Dataset						
			XUAMP	DRAMP	LAMP	dbAMP	APD3	YADAMP	CAMP
ESM2	t12	LoRA	0.482 (0.005)	0.541 (0.003)	0.766 (0.005)	0.809 (0.007)	0.820 (0.007)	0.926 (0.004)	0.945 (0.007)
	t12	QLoRA	0.468 (0.003)	0.547 (0.005)	0.760 (0.003)	0.799 (0.006)	0.837 (0.008)	0.928 (0.006)	**0.981 (0.007)**
	t33	LoRA	0.532 (0.006)	0.586 (0.003)	0.792 (0.007)	0.845 (0.008)	0.852 (0.008)	0.944 (0.005)	0.957 (0.012)
	t33	QLoRA	0.533 (0.004)	0.598 (0.005)	0.798 (0.005)	0.843 (0.008)	0.850 (0.009)	0.950 (0.006)	0.964 (0.004)
	t36	LoRA	**0.544 (0.005)**	0.599 (0.005)	0.802 (0.009)	0.847 (0.007)	**0.870 (0.002)**	0.945 (0.005)	0.963 (0.004)
	t36	QLoRA	0.525 (0.006)	**0.616 (0.003)**	0.804 (0.008)	0.841 (0.011)	0.870 (0.007)	0.958 (0.006)	0.970 (0.004)
	t48	QLoRA	0.533 (0.015)	0.605 (0.006)	**0.826 (0.004)**	**0.852 (0.020)**	0.862 (0.009)	0.947 (0.005)	0.968 (0.007)
ProtT5	xl	LoRA	0.521 (0.013)	0.607 (0.006)	0.820 (0.007)	0.839 (0.010)	0.857 (0.007)	**0.957 (0.006)**	0.960 (0.006)
	xxl	LoRA	0.526 (0.011)	0.602 (0.011)	0.817 (0.007)	0.843 (0.007)	0.868 (0.003)	0.956 (0.008)	0.970 (0.009)

ROC curves

Figure S12 shows the Receiver Operating Characteristic (ROC) curves for various models evaluated on the LAMP, dbAMP, APD3, YADAMP and CAMP datasets. For each dataset, the panel includes the ROC curves for various ESM2 models and the ProtT5xl model, fine-tuned using LoRA or QLoRA. The ROC curves represent the average ROC curve for five runs using different random seeds for the fine-tuning of the models. For comparison purposes, we additionally include the ROC curve for an SVM classifier trained on embeddings of the ESM2t48 model. We also provide in the legend the AUC values.

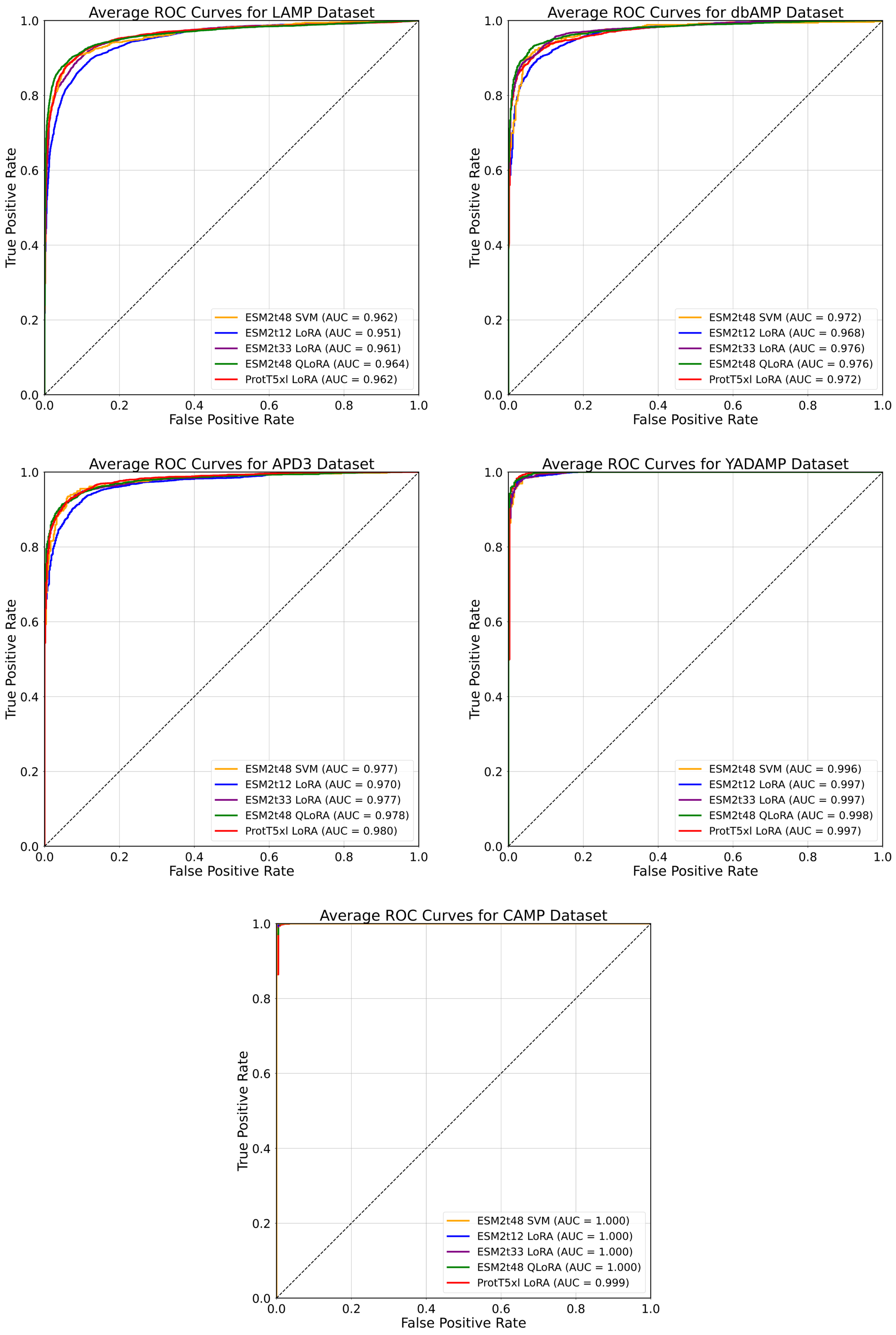

Figure S12. ROC curves for models evaluated on each dataset. We compare the SVM classifier trained on the ESMt48 models embeddings (orange) with ESM2t12 (blue), ESM2t33 (purple), ESM2t48 (green), as well as ProtT5xl (red) fine-tuned with LoRA or QLoRA.

Effect of Training Dataset Size on Model Performance

To assess model robustness and performance under varying data availability, we conducted experiments using progressively smaller subsets of the training data. Specifically, we evaluated model performance at 12.5%, 25%, 50%, and 100% of the full training set for both the DRAMP and XUAMP datasets. We evaluated both the embedding-based transfer learning and LoRA fine-tuning approaches. For each approach, we tested representative models of varying sizes, including ESM2t12, ESM2t33, ESM2t36, and ProtT5xl. Figures S13 and S14 present the classification performance (ROC AUC) of each method under decreasing training data availability. All experiments were repeated five times with different random seeds.

The results highlight the overall stability of transfer learning approaches across varying training data sizes, underscoring their effectiveness in scenarios of low labeled data availability. As expected, the performance generally declines with reduced training data. Notably, the LoRA fine-tuning method exhibits slightly larger average ROC AUC declines of 0.9%, 2.5%, and 4.4% for data reductions to 50%, 25%, and 12.5%, respectively, compared to the 0.6%, 1.6%, and 3.0% declines observed for the embedding-based approach. Despite this difference in relative decline, the absolute performance of the LoRA fine-tuned models remains higher than that of the embedding-based approach at every data fraction. As a final remark, we did not observe any consistent correlation between the magnitude of this decline and the underlying model size.

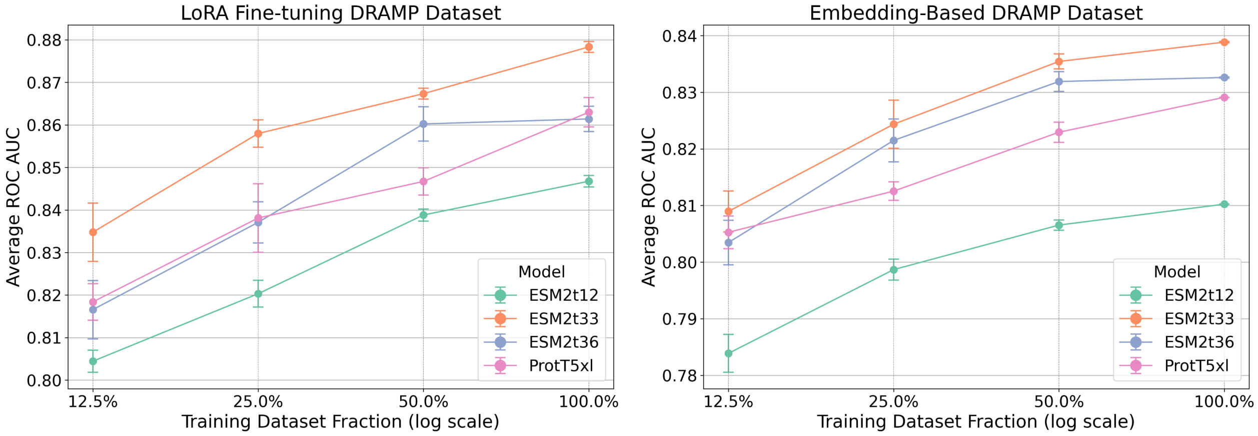

Figure S13. ROC AUC scores for the DRAMP dataset across varying training data fractions, for LoRA fine-tuned models (left) and embedding-based transfer learning with LogReg classifier (right). Each point represents the mean ROC AUC over five runs, with vertical lines indicating standard error.

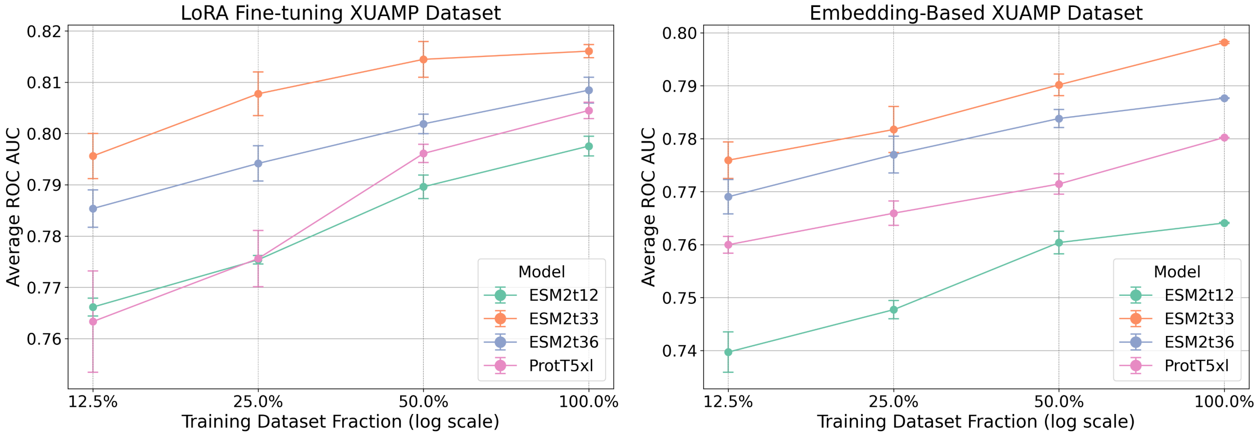

Figure S14. ROC AUC scores for the DRAMP dataset across varying training data fractions, for LoRA fine-tuned models (left) and embedding-based transfer learning with LogReg classifier (right). Each point represents the mean ROC AUC over five runs, with vertical lines indicating standard error.

References

[1] Xu, J. *et al.* Comprehensive assessment of machine learning-based methods for predicting antimicrobial peptides. *Briefings in bioinformatics* **22**, bbab083 (2021).

[2] Yan, K., Lv, H., Guo, Y., Peng, W. & Liu, B. sAMPpred-GAT: Prediction of antimicrobial peptide by graph attention network and predicted peptide structure. *Bioinformatics* **39**, btac715 (2023).

[3] McInnes, L., Healy, J. & Melville, J. UMAP: Uniform manifold approximation and projection for dimension reduction. *arXiv preprint arXiv:1802.03426* (2018).

[4] Hu, E. J. *et al.* LoRA: Low-rank adaptation of large language models. *arXiv preprint arXiv:2106.09685* (2021).

[5] Schmirler, R., Heinzinger, M. & Rost, B. Fine-tuning protein language models boosts predictions across diverse tasks. *Nature Communications* **15**, 7407 (2024).

[6] Dettmers, T., Pagnoni, A., Holtzman, A. & Zettlemoyer, L. QLoRA: Efficient finetuning of quantized llms. *Advances in Neural Information Processing Systems* **36** (2024).
